# The Progress and Perspective of Organic Molecules With Switchable Circularly Polarized Luminescence

**DOI:** 10.3389/fchem.2020.00458

**Published:** 2020-06-12

**Authors:** Yang Gao, Can Ren, Xiaodong Lin, Tingchao He

**Affiliations:** College of Physics and Optoelectronic Engineering, Shenzhen University, Shenzhen, China

**Keywords:** circularly polarized luminescence, asymmetrical emission factor, organic material, multiple stimuli, reversible switch

## Abstract

Circularly polarized luminescence (CPL) has been under intense research for future applications in high-resolution 3D displays, smart sensors, and information technologies. Different types of CPL materials have been developed, but neither the handedness nor the asymmetrical luminescence degree can be inferred from the material composition or the components. CPL materials with switchable handedness or emission wavelength play an important role, reducing the need for repetitive bottom-up synthesis. Here, we have presented switchable CPL behaviors toward multiple reported stimuli, including light irradiation, host–guest interaction, metal ions, pH, solvent, temperature, etc. This summary and discussion of the effective stimuli is aimed to promote rational future material exploration and boost related multidisciplinary applications.

**Graphical Abstract:**
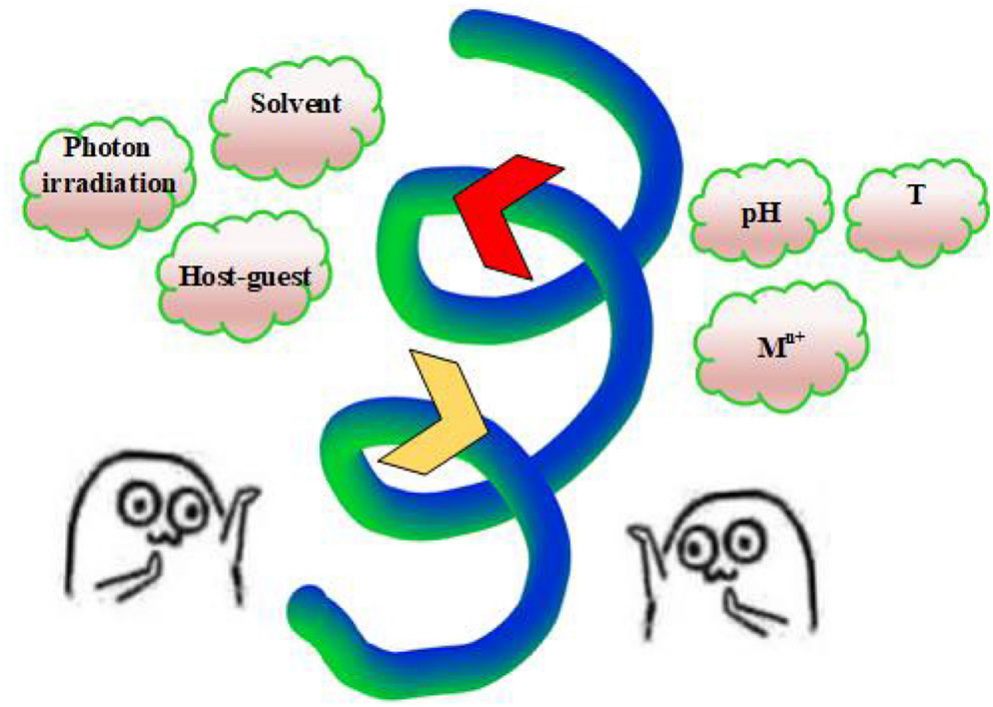
CPL materials with switchable handedness or emission wavelength toward multiple stimuli, including light irradiation, host-guest interaction, metal ions, pH, solvent, temperature, etc.

## Introduction

Circularly polarized luminescence (CPL) has been the subject of intense research due to its possible applications in new photonic/photoelectronic devices (Han J. et al., 2018; Zhang et al., 2020), smart sensors, high-resolution 3D displays, information technologies, etc. A variety of CPL materials have been developed to date, including rare-earth metal-based coordination complexes and organic and inorganic molecules/assemblies. Organic luminescent materials have enjoyed a major role in their development due to the wide range of possible structural components, moderate to high emission efficiency, and multiple intra-/inter-molecular interaction modes.

Although there are a number of known strategies to design CPL active materials and a large library of material structures are available (Pop et al., 2019; Sang et al., 2019; Zhao W. L. et al., 2019; Ouyang and Liu, 2020), it is as yet unrealistic to predict related CPL activities for any given structure. Neither the handedness nor the degree of asymmetry can be confidently or accurately predicted from the material composition or the components (e.g., enantiomer excess value). Thus, CPL materials with switchable emission characteristics have been the subject of intense research with the aim of obtaining strong CPL with selective handedness/emission wavelength. Recently, a number of novel approaches have been reported including facile applicable triggers, multiple emissive states, and high-quality emitters. For example, a helical structure incorporating pyrene units showed strong CPL in solution (g_lum_~10^−2^) with handedness, which was invertible by changing the solvent from toluene to DMSO (Takaishi et al., 2020). Also, a switch from circularly polarized fluorescence to ultra-long phosphorescence was achieved for a chiral carbazole phosphor (Li et al., 2020).

Though there have been some recent partial reviews (Ma J. L. et al., 2019; Sang et al., 2019), CPL emitters whose emission is switchable under various stimuli have not been comprehensively reviewed. To fill this gap, a review of switchable CPL behavior is presented herein, including the use of irradiation with light, host–guest interaction, metal ions, pH, temperature, and solvents as stimuli. The discussion will be focused on reversible behavior. Also, a brief explanation of how CPL measurements are performed is included. Hopefully, this review will help promote the design and exploration of future materials and boost development of related multidisciplinary applications.

## Measurement

To describe CPL materials properly, a number of parameters related to both emission and polarization are involved. Concerning emission behaviors, features usually mentioned include the type of emission (fluorescence, phosphorescence), the emission wavelength (λ_em_), quantum efficiency (Φ), and the emission lifetime (τ). To characterize spectroscopic features of chiral materials, circular dichroism (CD) and CPL spectra are often used to study the chirality in the ground and excited states, respectively. In the CD measurement, alternative left- and right-handed light beams pass through the chiral medium, which show different light propagation speeds. Different molar CD (Δε) can be recorded, and the asymmetric factor of CD can be calculated according to

(1) $$\begin{array}{l} g_{abs}=\frac{\Delta \varepsilon }{\varepsilon }=2\times \frac{\varepsilon_{L}-\varepsilon_{R}}{\varepsilon_{L}+\varepsilon_{R}} \end{array}$$

where ε_L_ and ε_R_ represents the extinction coefficients for left- and right-handed circularly polarized light, respectively.

The CPL measurement utilizes fluorescence spectrometry with additional compartments for the polarization detection. Usually the excitation beam is polarized with a polarizer before entering the sample, and a modulated circular polarizer is applied after the emission beam to obtain the separate intensity of the left-handed and right-handed CPL. The level of circular polarization in emission is termed as the dissymmetry or emission g-factor, which is formulated as followed:

(2) $$\begin{array}{l} g_{lum}=\frac{\Delta I}{I}=2\times \frac{I_{L}-I_{R}}{I_{L}+I_{R}} \end{array}$$

The theoretical range of g_lum_ is from −2 to +2. For organic molecules in solution, the g_lum_ value appears usually in the range of 10^−5^~10^−3^. While in aggregated state or in condensed state, the value increases to 10^−3^~10^−1^.

A switch of the CPL behaviors will be discussed in the context of the aforementioned parameters toward various stimuli.

## Irradiation With Light

**Figure 1:**
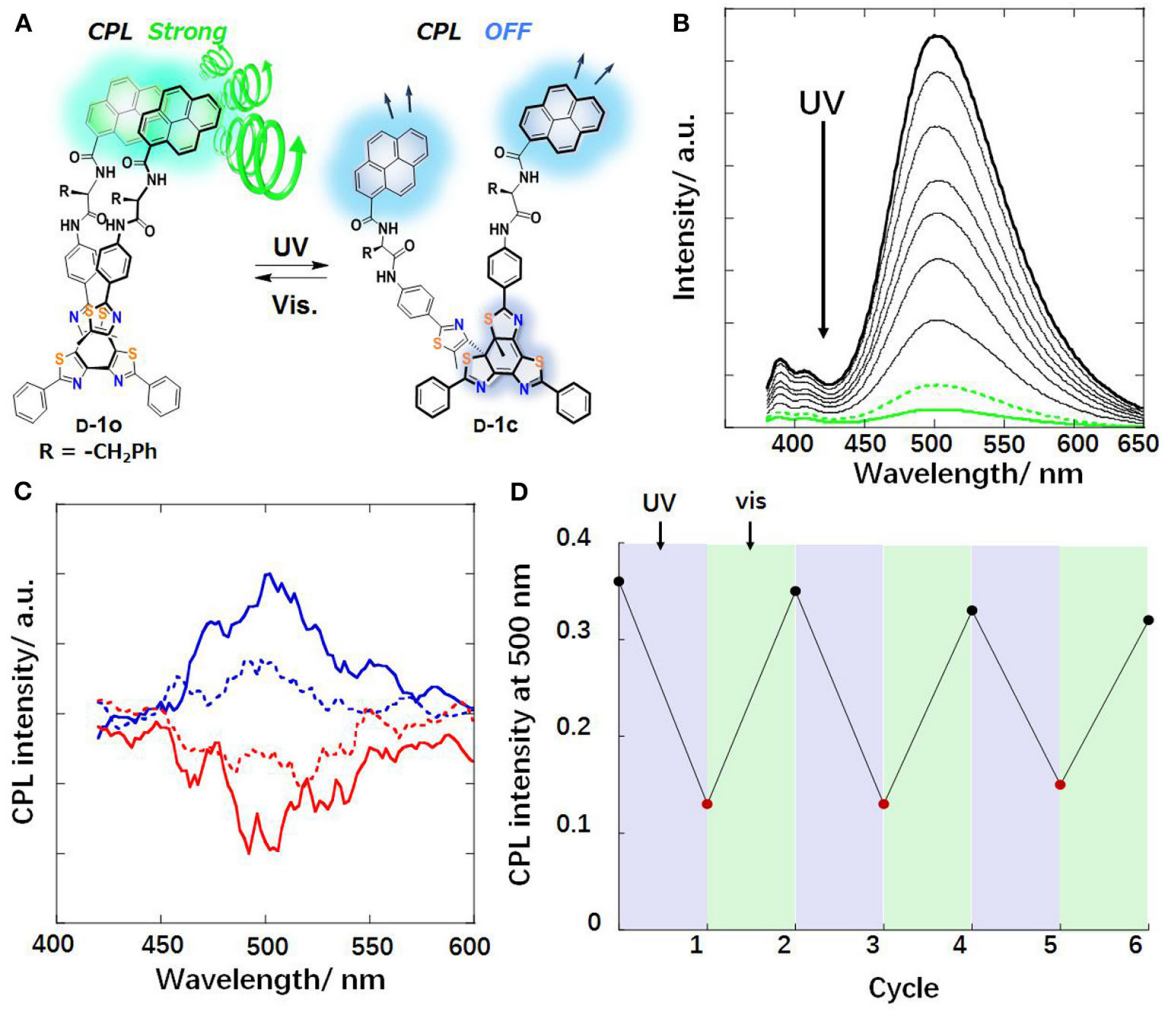
**(A)** Chemical and CPL change of pyrene stack with tetrathiazole D-1 toward light irradiation. **(B)** Fluorescence spectral change of L-1o upon UV irradiation $\left(4.6\times 10^{-6}M,CHCl_{3}\right)$. Solid thick black line: 1o-form, solid green line: 1c-form, dashed green line: at photo stationary state (PSS), other traces were recorded at an irradiation interval of 5 s. **(C)** CPL spectra of 1o $\left(1.7\times 10^{-4}M,CHCl_{3}\right)$. D-form: upper solid blue line; L-form: lower solid red line; dashed lines: at PSS. **(D)** Reversible CPL intensity changes of D-1o (500 nm, CHCl_3_) (Reproduced with permission; Hashimoto et al., 2016). Copyright 2016, Royal Society of Chemistry.

**Figure 2:**
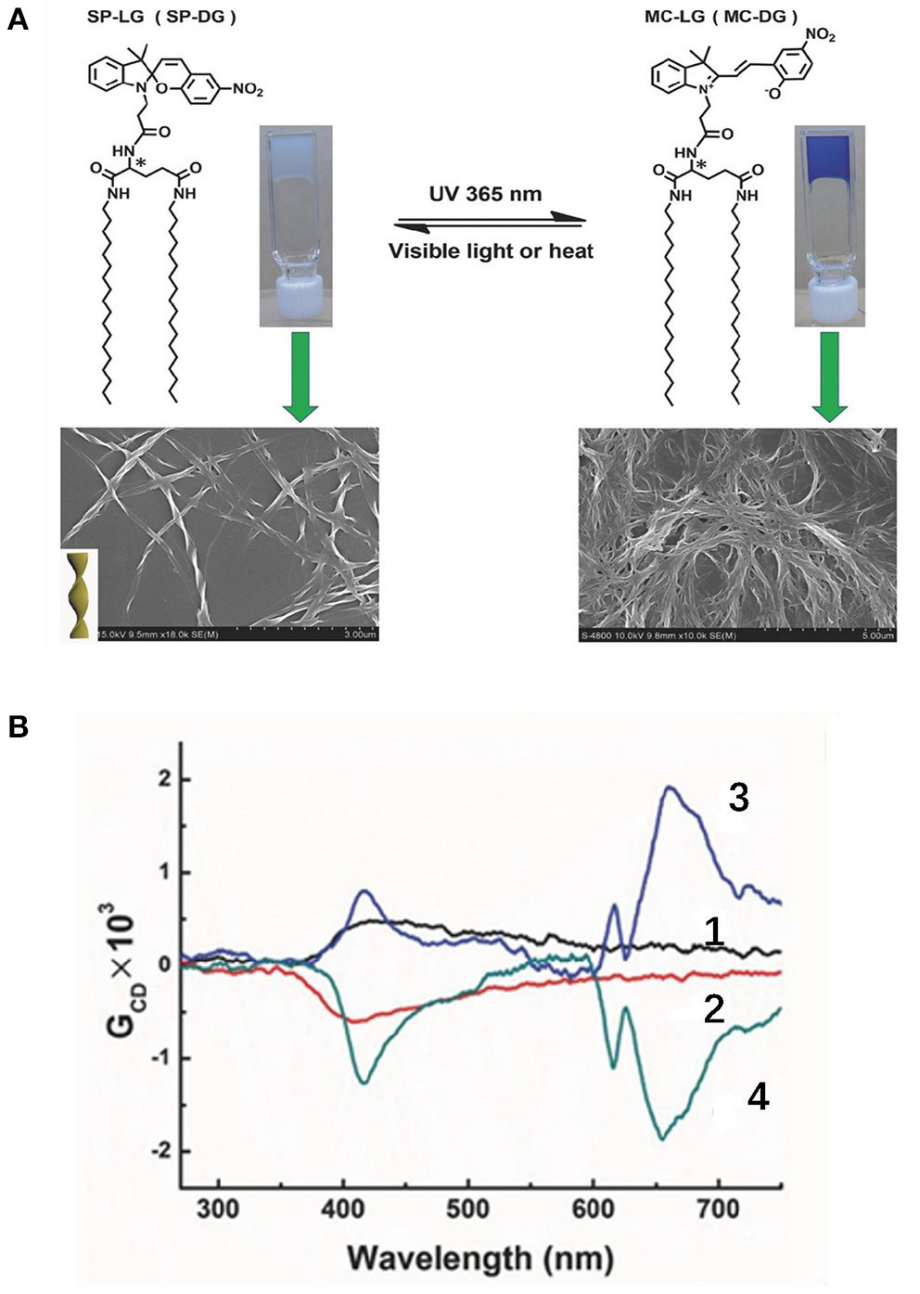
**(A)** Reversible molecular structure change of spiropyran-modified glutamate gelator between a colorless closed ring state and a blue zwitterionic emissive state toward alternating UV and visible light irradiation, with the related SEM images of SP-LG xerogels (lower part). **(B)** CPL spectra before UV irradiation (SP-LG (1), SP-DG (2) gels) and after UV irradiation for 8 min (MC-LG (3), MC-DG (4) gels). Reproduced with permission (Miao et al., 2017). Copyright 2017, John Wiley and Sons.

Irradiation with light can influence the CPL behaviors through photo cyclization/de-cyclization, photo induced isomerization, and selected population of specific excited state. Moreover, photoreactions are usually induced by UV radiation and reversed by lower-energy visible light or heat treatment. An on-off switch based on photo cyclization was observed for a photochromic tetrathiazole attached pyrene dye (Hashimoto et al., 2016). When the individual pyrene units were attached via a chiral phenylamine spacer to a tetrathiazole core, the π-π stacking of two phenylthiazoles resulted in a helical conformation of the core, and two pyrene units were arranged in close proximity. Thus, an intramolecular pyrene excimer was formed (Figure 1), and a CPL signal at 500 nm with a large |g_lum_| (0.01) was observed. When the helical conformation of the photochromic core was destroyed by UV-light driven cyclization, the pyrene units were separated from each other, and the CPL was quenched. A reversible off-on CPL switch was observed for enantiomeric glutamate gelators modified with a spiropyran moiety (Figure 2) upon alternated UV and visible irradiation (Miao et al., 2017). Chirality transfer from the chiral glutamate part to the luminophore was facilitated in the gel state. Upon UV irradiation (365 nm), the spiropyran unit changed from a colorless closed ring form to a blue zwitterionic merocyanine state accompanied by a red CPL signal (662 nm). After exposure to visible light, the CPL phenomenon was suppressed. This reversible process worked for over 30 cycles when applied in a re-writable printing application.

**Figure 3:**
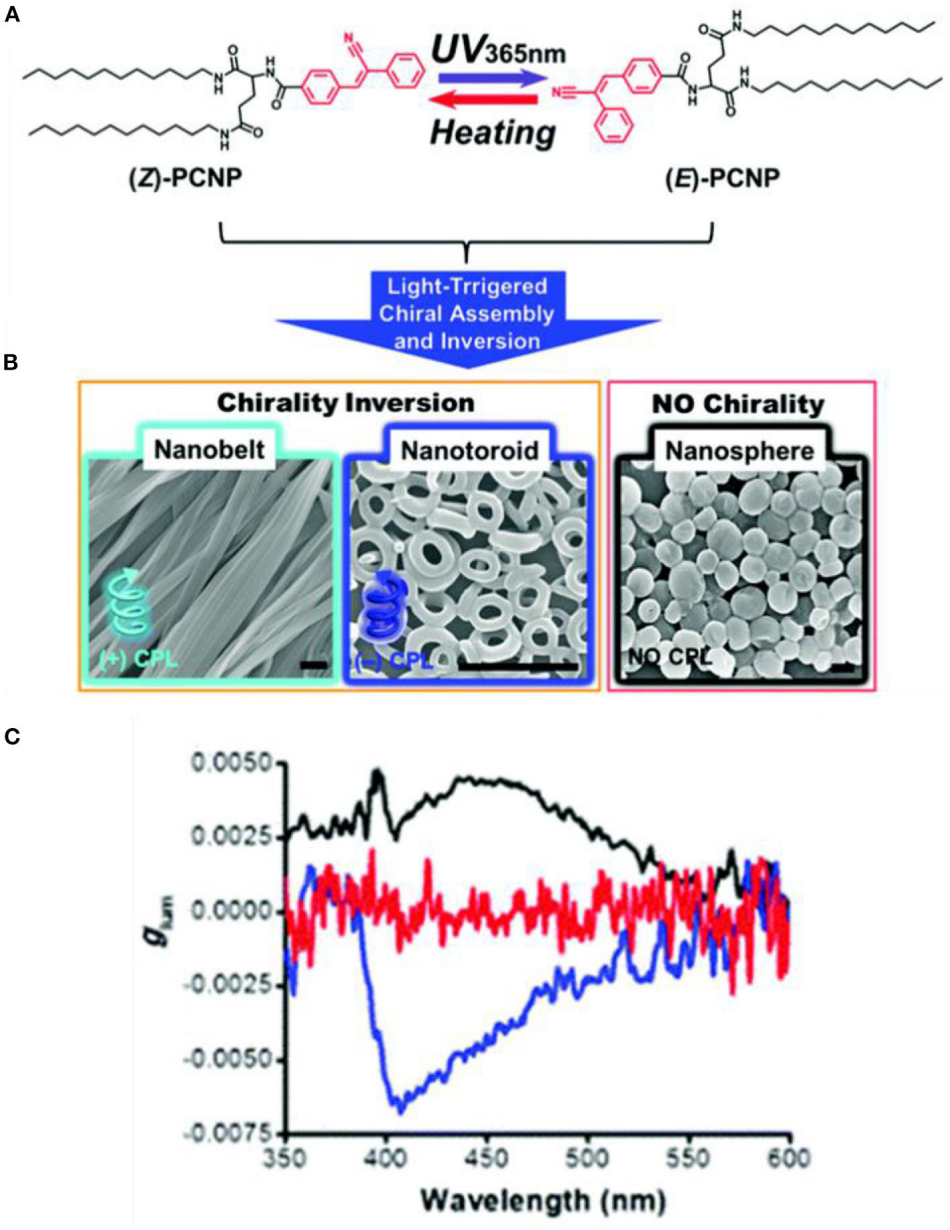
**(A)** Z-E isomerization of PCNP under UV irradiation. **(B)** SEM images of the different assembled nanostructures with different irradiation duration (365 nm). From left to the right: Nanobelts (0 min); nanotoroids (15–120 min); and nanospheres (150 min). Scale bar: 1 mm. **(C)** Glum curves of nanobelts (black), nanotoroids (blue), and nanospheres (red). Reproduced with permission (Jin X. et al., 2018). Copyright 2018, Royal Society of Chemistry.

**Figure 4:**
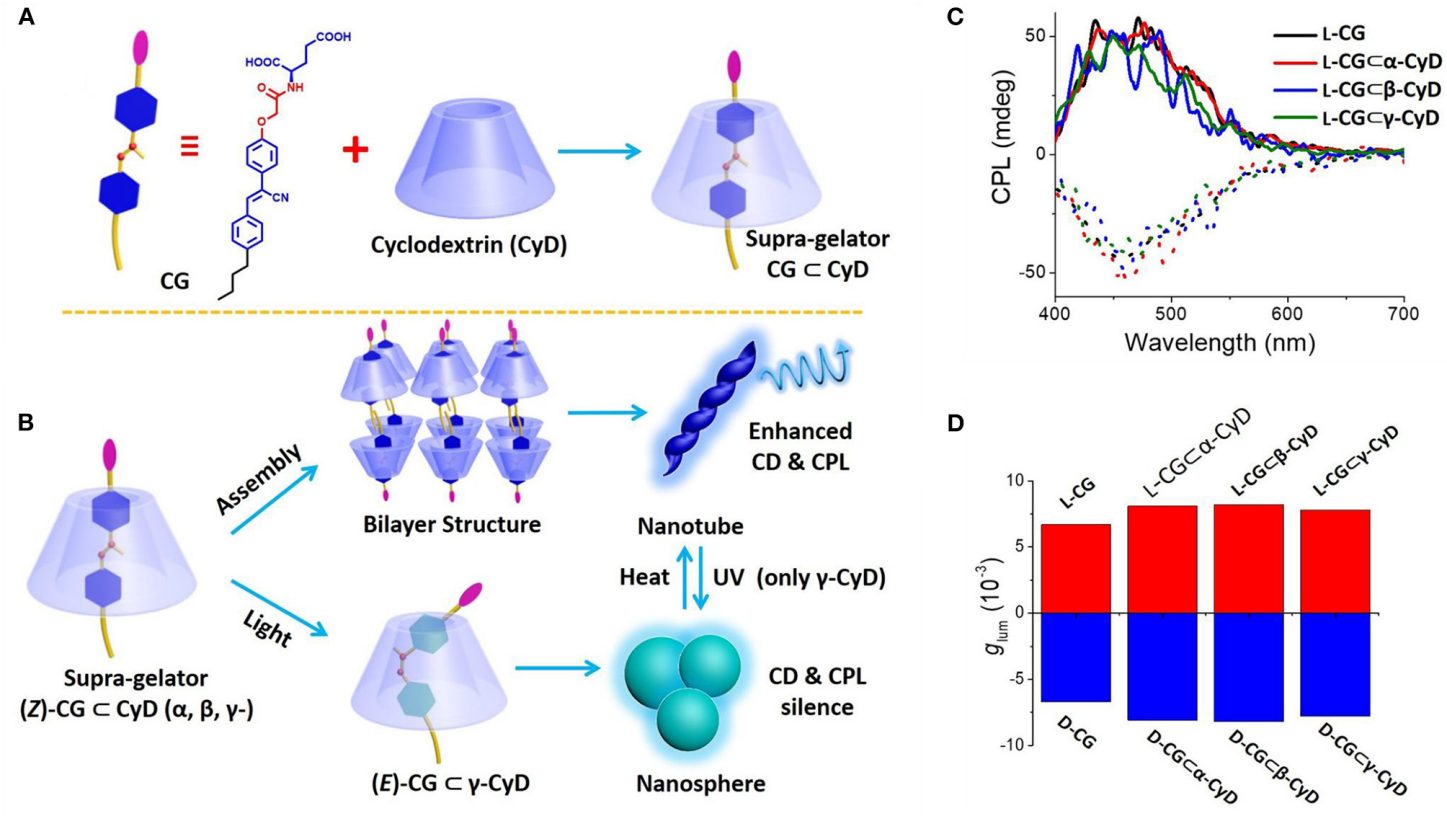
**(A)** Molecular structure of hydrogelator CG and schematic formation of supra-gelator CG-CyDs. **(B)** Suggested gelation route of supra-gelator CG-CyDs (upper), UV irradiation induced Z–E isomerization in CG-CyD and the morphology change between nanotube and nanosphere (bottom). **(C)** CPL curves of CG and CG-CyDs hydrogels. **(D)** Glum values plot of CG and CG-CyDs hydrogels. The dashed lines refer to the counterparts of the D-configuration CG based gels. [CG] = 6.5 mM, [CG-CyD] = 6.5 mM. Reproduced with permission (Ji et al., 2019). Copyright 2019, Royal Society of Chemistry.

Light irradiation induced *Z-E* isomerization of a cyanostilbene-based chromophore resulted in different assembled structures and different related CPL behaviors. Upon exposure to UV-light, cyanostilbene-conjugated glutamide (Figure 3) assembled into different morphologies with inversed CPL sign (Jin X. et al., 2018). The rigid planar (Z)-PCNP formed nanobelts with a lamellar structure, exhibiting left-handed CPL (g_lum_ ~4.5 × 10^−3^). Upon irradiation at 365 nm, co-existing rigid Z- and flexible E- isomers assembled to form nanotoroids, which showed right-handed CPL (g_lum_ ~6.6 × 10^−3^). Similarly, a host–guest supra-gelator, which was formed by encapsulating a chiral Z-cyanostilbene gelator (CG) inside the cavity of γ-cyclodextrin (CD) (Figure 4), showed reversible off-on CPL, which could be switched using UV-irradiation and heating (Ji et al., 2019). The super-gelator assembled into a bilayer structure and showed CPL signal at 450 nm with slightly enhanced g_lum_ value (~7.9 × 10^−3^). The handedness of the super-gel followed the chirality of the CG unit. When the super-gelator was subjected to UV irradiation, isomerization of Z-CG to E-CG took place, the gel collapsed to a suspension of nanospheres, and the CPL signal gradually disappeared. After heating the suspension to form a solution and natural cooling down, the supra-gel was reproduced with the same CPL activity. Moreover, the reversible process was pretty robust, as confinement of the CG unit inside the cavity of γ-CD make it resistant to fatigue.

**Figure 5:**
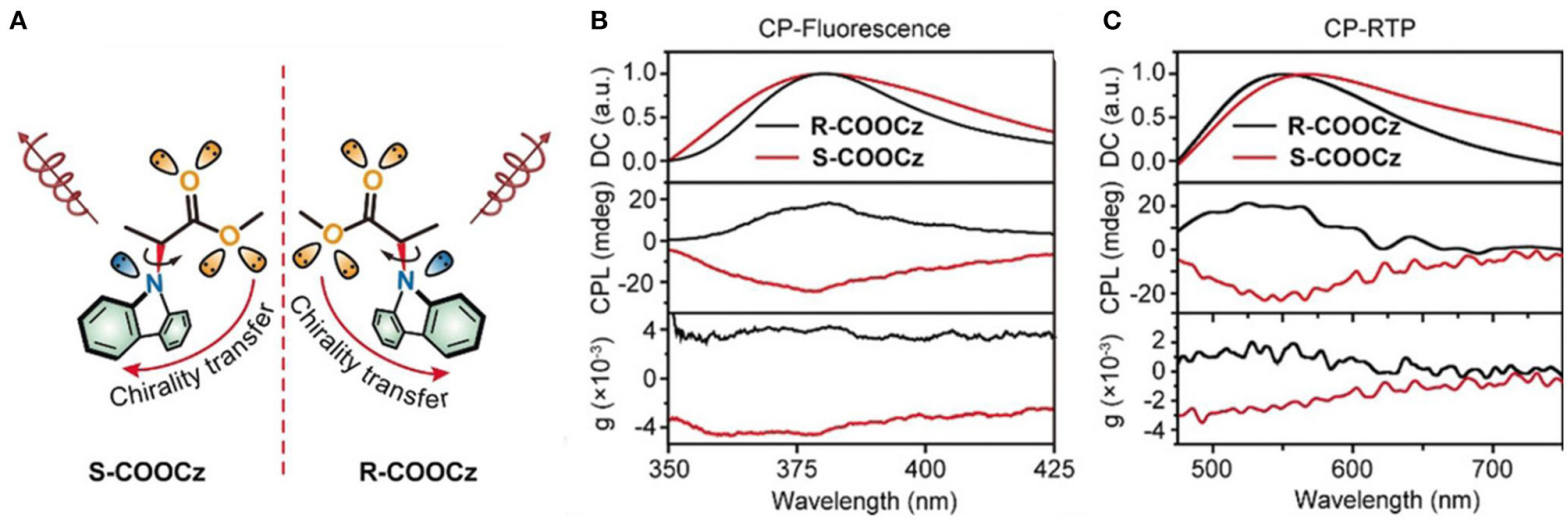
**(A)** Molecular design of chiral CP-OURTP molecules (S- and R- COOCz). **(B)** CP-Fluorescence curves excited at 295 nm. **(C)** CP-RTP curves excited at 365 nm. Reproduced with permission (Li et al., 2020). Copyright 2020, John Wiley and Sons.

Besides the above photo chemical mechanism, the CPL switch was also observed for the photophysical mechanism. Light irradiation at selected wavelength can populate specific excited states, select the emissive state energy, then manipulate the emission probability and the emission lifetime. When a chiral ester chain was linked to the N-position of a carbazole phosphor (Li et al., 2020), an H-type aggregate in the condensed state showed CPL at 369 and 379 nm (g_lum_ ~0.0031) upon photoexcitation at 365 nm. Moreover, chiral phosphorescence at 550 and 596 nm (g_lum_ ~0.0027) was observed at room temperature (CP-RTP) after removal of the photo excitation (Figure 5). With extended irradiation time (365 nm, <40 s), ultralong lifetime CP-RTP (CP-OURTP) was observed by naked eye at room temperature or at low temperature. With elevated temperature at 50°C, the signal of CP-OURTP diminished within 5 min. After lowering the temperature and repeating photo-activation, the CP-OURTP signal recovered without photo bleaching. Thus, a reversible irradiation turn-on and thermal turn-off CP-OURTP system was developed.

## Host–Guest Interaction

Engineered rotaxanes with different sizes were demonstrated as containers to encapsulate various guests. Association–disassociation of the host–guest interaction can serve as a switch for further excimer formation, chirality transfer, chirality inversion, and modulate the on-off state of CPL behaviors. When two polycyclic aromatic chromophores (PAH) (NDI, Pyrene, perylene, and fluorene) were linked to the same face of modified crown-ethers, the close geometrical contact between the PAH units resulted in the formation of intramolecular excimers (Homberg et al., 2018). G_lum_ values as high as ~10^−2^ were observed. After selective binding the crown-ether with metal ions such as Na^+^ and Ca^2+^, the central cryptand acquired a new geometry in which the chromophores were situated far away from each other. The excimers were thus disrupted and the related CPL suppressed.

**Figure 6:**
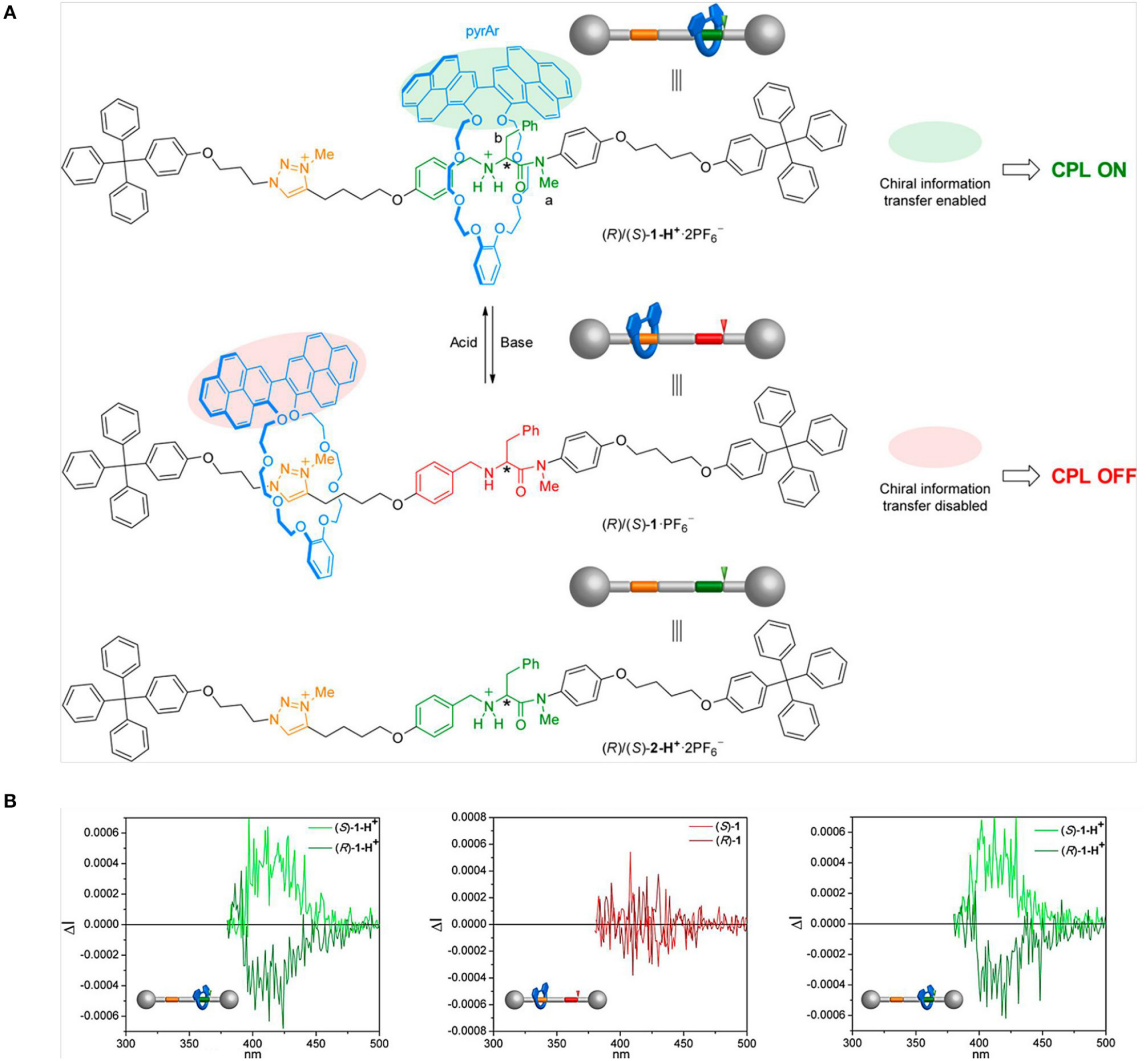
**(A)** On–off CPL switching of rotaxanes (R)/(S)-1-H^+^·2PF_6_ by acid/base- controlled chirality transfer from the thread to the pyrene containing macrocycle. **(B)** CPL spectra of 1-H^+^·2PF6^−^ (left), 1·PF6^−^ (middle), and 1-H^+^ (right), obtained by protonation of 1·PF6^−^ with CF_3_CO_2_H in CHCl_3_, condition: normalized ΔI scale, λ_**exc**_ = 355 nm, ca. 1 × 10^−5^ M. Reproduced with permission (David et al., 2019). Copyright 2019, American Chemical Society.

When the host was changed to chiral (P-/M-) 2,6-helic[6]arene cycle, the complexation with guest 4-[(4′-N, N-diphenylamino)styryl]-N-methylpyridinium iodide in water showed mirror-imaged CD and CPL signals (Guo et al., 2019). The chirality transfer from the host to the guest resulted in chiral emission of the guest, which was absent for the guest alone. Moreover, the chirality transfer was tunable by temperature and pH. In detail, higher temperature (up to 70°C) caused faster rotation and motion of both the host and the guest, then deteriorated the chirality transfer. Similarly, either lower or higher pH other than the neutral condition caused lower CPL signals due to either destructed assembly or weakened host–guest interaction. By selecting different inclusion units for a [2]rotaxane, David et. al reported a molecular machine based on-off CPL switch (David et al., 2019). A crown-ether macrocycle with an emitting 2,2′-bipyrene unit was threaded with a secondary ammonium unit, which was linked with chiral D- or L- phenylalanine moieties (Figure 6). When H-bonding was formed between the luminophore and the chiral center under acidic conditions, CPL signals appeared and the handedness was determined by the phenylalanine unit. By contrast under basic conditions, the location of the macrocycle shifted along the thread to the triazolium group, which disabled the aforementioned chirality transfer. Such an on-off switch worked *in-situ* and only had impact on the CPL signal without quenching the photoluminescence.

In a similar concept, alternative addition of potassium ions (K^+^) and cryptand were applied to invert the chirality of a G-quadruplex DNA (G4 DNA) (Chen et al., 2019). Parallel arrangement G4 DNA can be changed to anti-parallel arrangement by addition of K^+^, and the inclusion of luminescent ThT (3,6-dimethyl-2-(4-dimethylaminophenyl)-benzo-thiazolium) cations produced different handedness (490 nm, g_lum_ ~0.5 × 10^−3^ to 1.5 × 10^−3^, 10°C). Moreover, the ordered assembly of G4 DNA and ThT was promoted at low temperature (10°C), which could switch CPL on. Higher temperature (10–70°C) dissolved the assembly and turned the CPL off. Thus, switching of both handedness and intensity was realized for the assembled complex of G4 DNA and ThT.

## Metal Ions

Metal-ligand interaction has been demonstrated as a facile method to tune CPL performance, by modifying the active chromophores through changes in their conformation, chemical composition, electronic structure, assembly behavior, etc. This is often reversible by extracting the metal ion with strong chelating ligands. Multiple metal ions have been applied, including Zn^2+^, Ag^+^, Ni^2+^, and Al^3+^.

**Figure 7:**
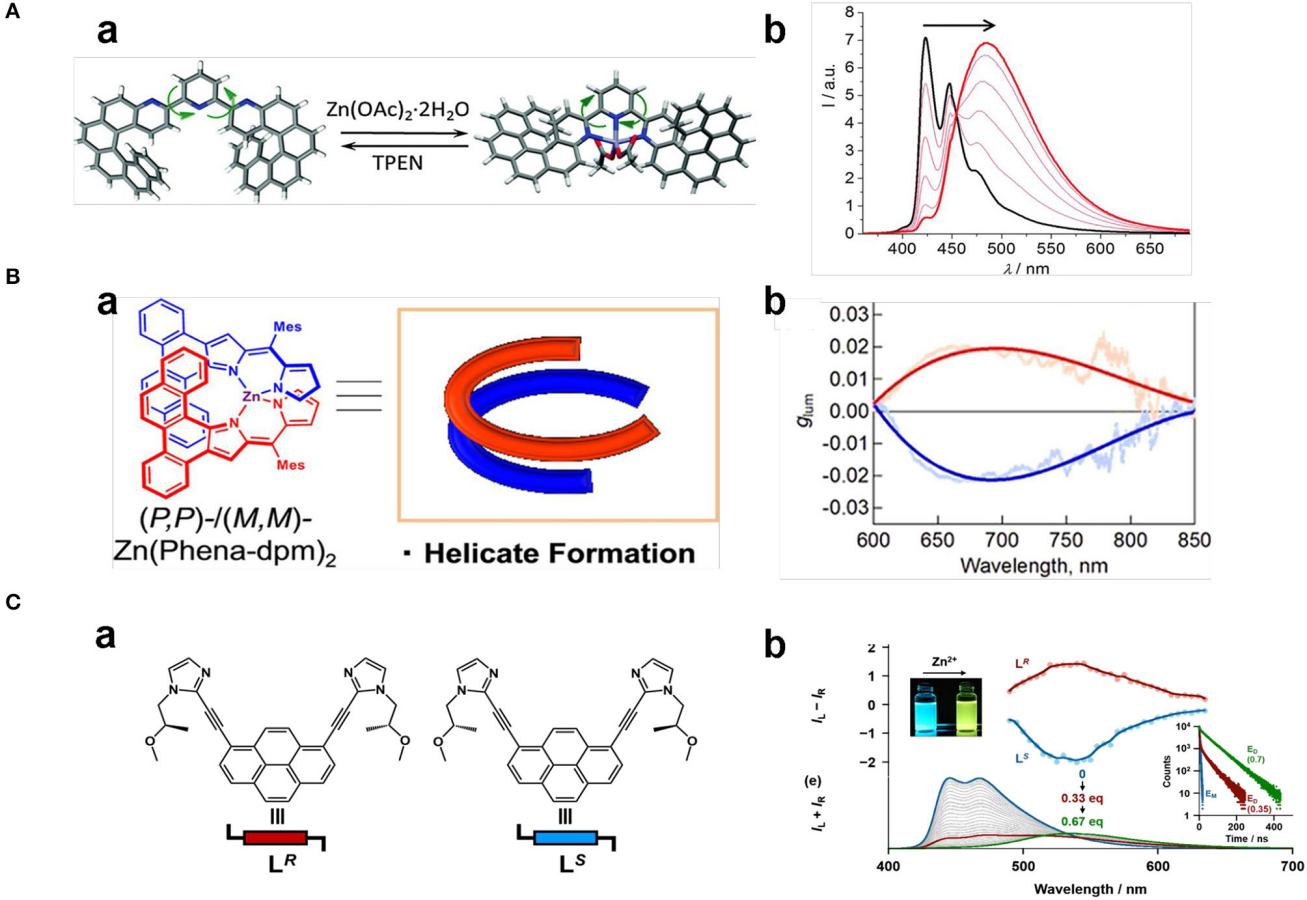
**(A)** (a) Reversible Zn(II) complexation–decomplexation process of (P,P)-1 upon addition of Zn(OAc)_2_ and TPEN. (b) Complexion-related fluorescence change of (P,P)-1 (λ_ex_ = 350 nm, 2.2 × 10^−5^ M in CH_2_Cl_2_. **(B)** (a) Zinc(II) helicate (Zn(Phena-dpm)_2_) formed by coordinating with two achiral phenanthrene-fused dipyrromethene ligands. (b) Smoothed g_lum_ curves of M,M (red line) and P, P enantiomer (blue line) measured in toluene (λ_ex_=580 nm), respectively. **(C)** (a) Chemical structures of chiral ligands L^R^ and L^S^. (b) Emission color change (upper left) and emission spectral change (lower left, λ_ex_ = 380 nm) of L^S^ toward Zn^2+^, CPL of L^R^ and L^S^ (middle) (2.0 × 10^−5^ M) in the presence of Zn^2+^ (1.4 × 10^−5^ M), emission decay profiles of L^S^ (2.0 × 10^−5^ M) (right) without Zn^2+^ (blue), with low ratio of Zn^2+^ (7.0 × 10^−6^ M (red), λ_em_ = 446 nm), and with high ratio of Zn^2+^ (1.4 × 10^−5^ M (green), λ_em_ = 535 nm). **(A)** Reproduced with permission (Isla et al., 2016). Copyright 2016, Royal Society of Chemistry). **(B)** Reproduced with permission (Ito et al., 2018). Copyright 2018, John Wiley and Sons. **(C)** Reproduced with permission (Imai and Yuasa, 2019). Copyright 2019, Royal Society of Chemistry.

Turn-on of CPL upon coordination of Zn^2+^ with terpyridine, salen, and dipyrromethene units is accompanied by geometrical changes. A terpyridine suspended bis- aza[6]helicenic unit changed from trans, trans-N,N orientation to cis, and cis-N,N orientation upon coordination with Zn^2+^ ions (Figure 7A), which resulted in the flip-over of one helicene moiety and charge-transfer from the helicene to the terpyridine part (Isla et al., 2016). Correspondingly, the positive CD signal at 341 nm decreased slightly and the emission shifted from 420 to 480 nm with greatly enhanced intensity (Φ_FL_ from 0.08 to 0.19), while *g*_lum_ values decreased slightly to +1.2 × 10^−3^ and −1.4 × 10^−3^ for (P, P) and (M, M)-isomers, respectively. Moreover, the Zn^2+^-terpyridine coordination was reversible by adding a competitive ligand N, N, N′, and N′-tetrakis(2-pyridylmethyl)ethane-1,2-diamine so that the CPL behavior could be recovered. BINOL based polymer enantiomers with Salen units showed turn-on CPL (465 nm, g_lum_ ~8 × 10^−3^) upon coordination with Zn^2+^ (0.3–4.0 equiv) (Meng et al., 2018), with the handedness being determined by the chiral BINOL unit. The CPL signal was induced by effective chirality transfer from the binaphthyl moiety to the Zn^2+^-Salen unit. Addition of EDTA as a competitive Zn^2+^ binder can switch-off the CPL signal. Another example of Zn^2+^ coordinated complex with turn-on CPL in the far-red region (wavelength: 700–850 nm, Φ_FL_: 0.23) was reported in 2018 (Ito et al., 2018). In this system, a pair of achiral benzo[a]phenanthrene-fused dipyrromethene ligands formed a helical structure upon coordination with Zinc(II) (Figure 7B), which showed strong exciton-coupled chiroptical responses in both ground state (g_abs_~0.20) and the excited state (g_lum_ ~0.022). Notably, the asymmetrical luminescence factor was the largest yet reported among rare-earth- and precious-metal-free small molecules.

**Figure 8:**
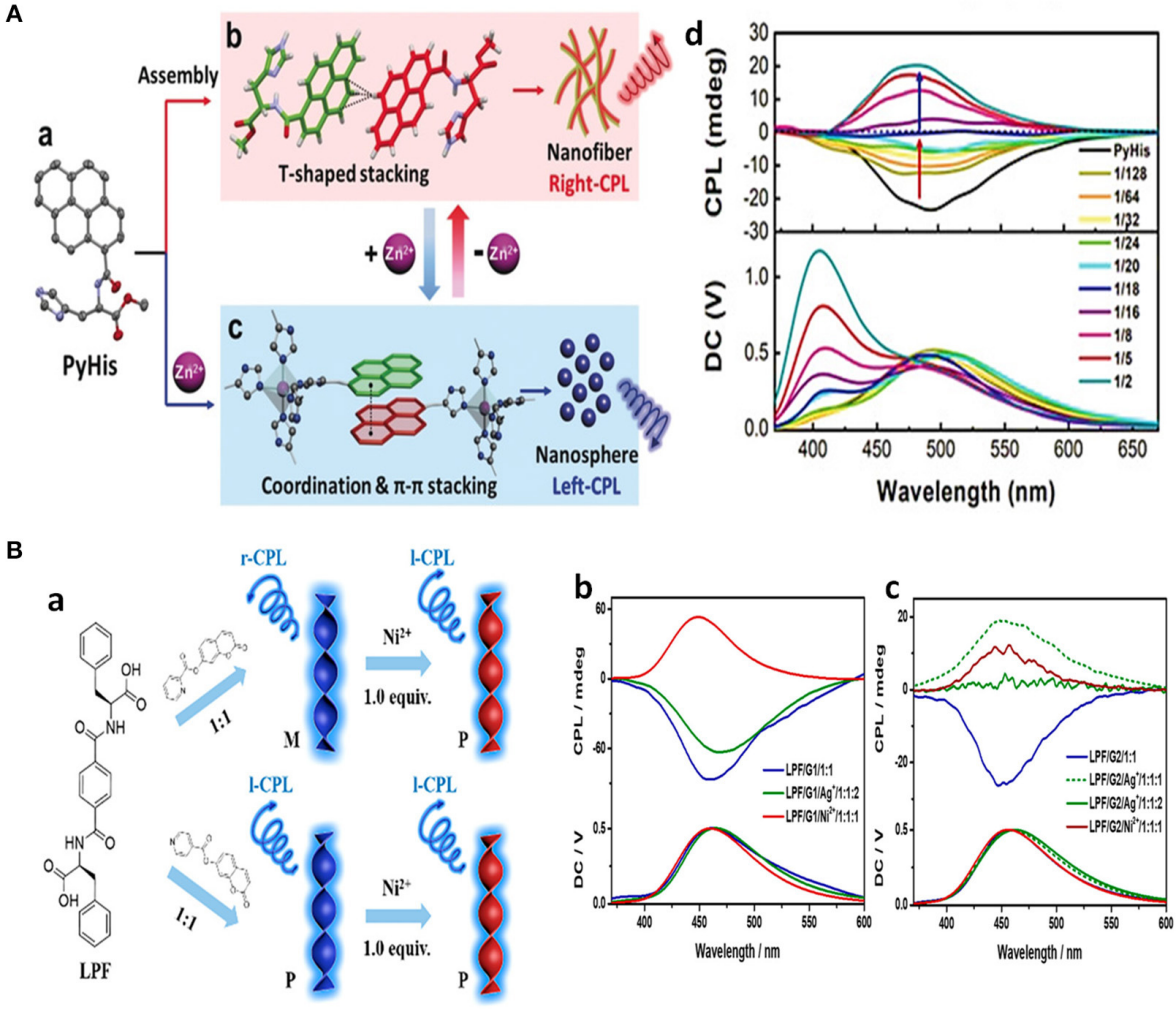
**(A)** Schematic illustration about assembly changes and CPL changes of the chiral gelator PyHis toward Zn^2+^. (a) Single-crystal structure. (b) T-shaped stacking of pyrenes in PyHis nanofibers, showing right-handed CPL. (c) π-π stacking of pyrenes with distorted triangular bipyramid [Zn(PyHis)_5_]^2+^ complex, which formed nanospheres with increasing amount of Zn^2+^ and showed left-handed CPL. (d) CPL spectra change of l-PyHis gel upon increased amount Zn^2+^ (λ_ex_=330 nm). **(B)** Schematic illustration of CPL behaviors dependent on pyridyl-N location of coumarin derivatives, and further Ni^2+^ (a). PL and fluorescence spectra of pyrenes of LPF/G1 (b), LPF/G2 (c) hydrogels with different amount of Ag^+^ or Ni^2+^ ions, excited at 320 nm. (a) Reproduced with permission (Niu et al., 2019). Copyright 2019, John Wiley and Sons. (b) Reproduced with permission (Wang et al., 2019). Copyright 2019, American Chemical Society.

Besides the on-off switch, a switch of the CPL emission colors were also reported for Zn(II) complexes in case of imidazole or histidine ligand, which originated from changes in their compositions or their assembly behavior. CPL activities of pyrene bridged dual chiral imidazole units (L^S^) were found to be dependent on the ratio of ligand to Zn^2+^ (Imai and Yuasa, 2019). When the molar ratio of [Zn^2+^]/[L^S^]_0_ was <0.33, a complexation with the formula of (Zn_1_L_3_)n appeared with blue pyrene monomer emission (445 nm, 467 nm), and no CPL signal was detected. When the molar ratio of [Zn^2+^]/[L^S^]_0_ increased to 0.33–0.67, a (Zn_2_L_3_)n complex formed that showed yellow emission (535 nm) (Figure 7C). The CPL signal was switched on, showing good asymmetrical behavior (|g_lum_| ~0.01) and emission quantum yield (Φ_FL_~0.10). By contrast, the ligand with L- chirality did not show a similar CPL switch. Pyrene-conjugated L-histidine assembled into nanofibers and formed gels in a mixture of EtOH/H_2_O (v/v 1:4), which exhibited right-handed CPL (500 nm). After complexation with Zn^2+^, the newly formed histidine-based penta-coordinated Zn(II) complex assembled into nanospheres, and the gel collapsed to form a suspension (Niu et al., 2019). Accordingly, the π stacking of the pyrenes unit shifted from a T-shaped to a nearly paralleled arrangement, the related CPL signal shifted from the excimer emission to the monomer emission, and the CPL handedness was inverted from right handedness to left handedness (Figure 8A). Such emission switch of wavelength and handedness was reversible by removing the Zn(II) ions with the strong chelator EDTA.

Ni^2+^ and Al^3+^ worked similarly through complexation to modify both the assembly behavior and CPL properties of organic molecules. A chiral phenylalanine-derived hydrogelator and a pyridine modified achiral coumarin co-assembled to form chiral gels, showing CPL at around 450 nm (g_lum_~10^−2^) with handedness being dependent on the position of the pyridine nitrogen atom (Wang et al., 2019). The introduction of Ni^2+^ removed the influence of the N-position and resulted in all the isomers producing the same P-helix and left-handed emission (Figure 8B). The different handedness was inferred to result from different dominant hydrogen-bonding interactions, including carboxylic acid-pyridine hydrogen bonds and amide hydrogen bonds. Thus, CPL with opposite handedness was obtained by tuning the nitrogen position and the metal additive. Aluminum ion (Al^3+^, AlCl_3_, ethanol) (Jin Q. et al., 2018) was found to turn-on the CPL of glutamide-derived amphiphilic Schiff base containing a 1-hydroxyl-2-naphthaldehyde group. Inhibition of photon-induced electron transfer (PET) or excited-state induced proton transfer (ESIPT) enhanced the blue emission and specific chirality transfer from the ligand to the chromophore resulted in observable CPL emission (465 nm, |${\text{g}}_{\text{lum}}|\sim 6.21\times 10{\;}^{-4}$).

**Figure 9:**
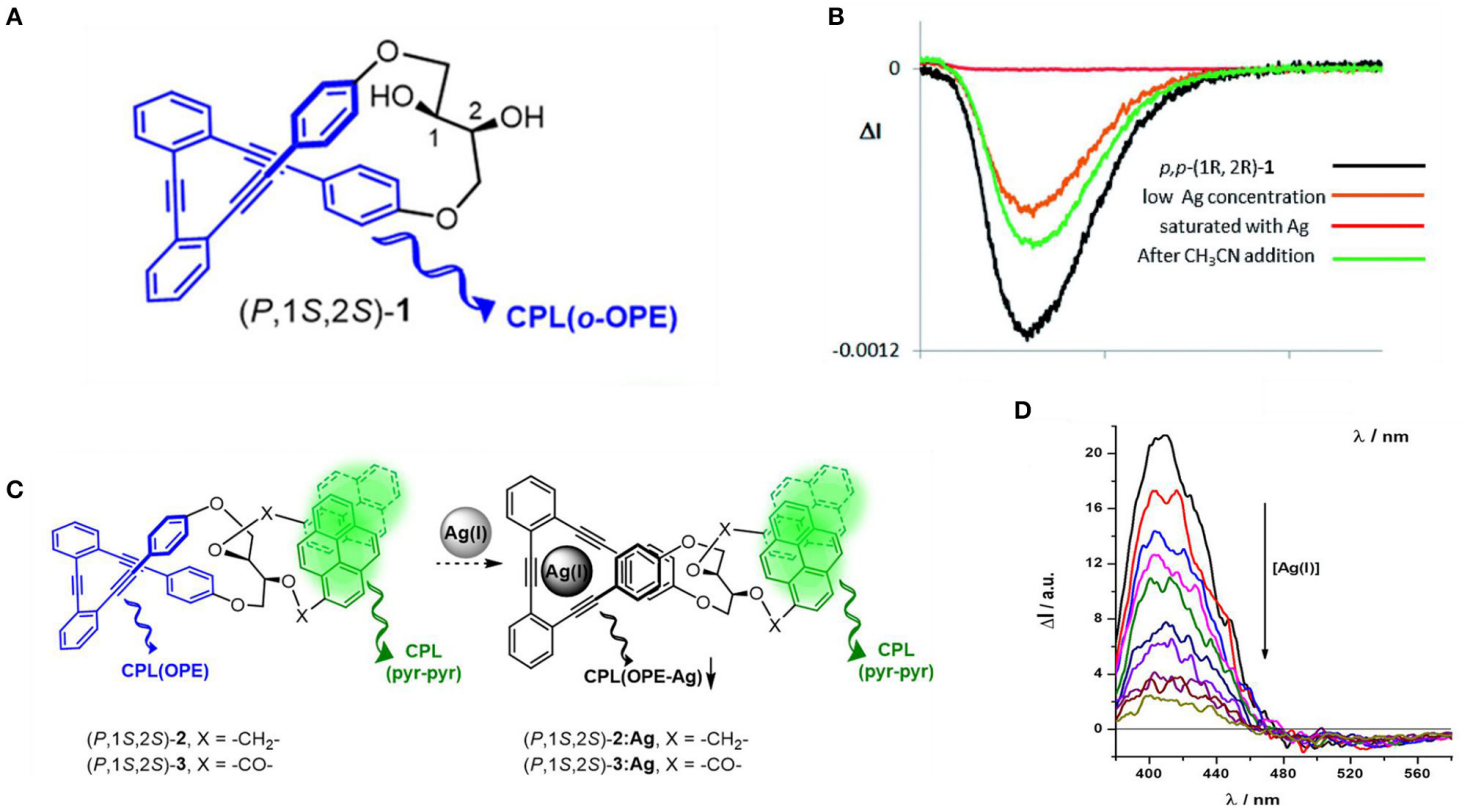
**(A)** Chemical structure of helical oligo(phenylethylene) (*P*,1s,2s)-**1**. **(B)** CPL titration curves with increasing amount of AgBF_4_ until saturation, and with following addition of CH_3_CN. **(C)** Schematic mechanism of a (*P*,1s,2s)-1 based ratiometric CPL probe based. **(D)** CPL titration curves of (*P*,1s,2s)-**2** with increasing amount of AgBF_4_. **(A,B)** Reproduced with permission (Morcillo et al., 2016). Copyright 2016, Royal Society of Chemistry. **(C,D)** Reproduced with permission (Reine et al., 2018). Copyright 2018, American Chemical Society.

Along with the coordination mechanism, metal ion also affected the assembly through carbophilic and oxophilic interactions. For instance, CPL activity of ortho-oligo-(phenylene)ethylene (OPE) foldmers can be tuned by adding either carbophilic Ag^+^ or oxophilic Ca^2+^, Sc^3+^, and Zn^2+^ ions. A series of ortho-oligo-(phenylene)ethylene (OPE) foldmers were confined into chiral helical structure by introducing enantiopure 2,3-dihydroxybutane diethers, exhibiting strong CPL responses (g_lum_ values up to 1.1 × 10^−2^) and a low but acceptable fluorescence efficiency (Φ_FL_ ~0.069 in CH_2_Cl_2_). When Ag^+^ was introduced, its strong affinity toward alkyne units resulted in its confinement within the OPE cavity. As a result, the conformation changed from a helical structure to a planar-like structure, which resulted in a decrease of g_lum_ values by up to one order of magnitude (Morcillo et al., 2016). Recovery of the asymmetrical optical properties can be achieved by adding a stoichiometric amount of competitive CH_3_CN to extract the confined Ag^+^ ions. When such an OPE helical skeleton was functionalized at both the C1 and C2 stereogenic positions with a pyrene unit, dual wavelength CPL emission in the blue (centered at 400 nm) and green (centered at 500 nm), originated from the OPE helicate and pyrene excimer located, respectively (Figure 9). Similar CPL silencing from the OPE unit upon carbophilic association with Ag^+^ ion was observed (Reine et al., 2018), while the CPL signal from the pyrene units was retained. With careful screening of the bridge between the chiral 2,3-dihydroxybutane diethers moiety and the pyrene units, the CPL signal [ΔI (400 nm)/ΔI (500 nm)] of one foldmer [(P,1S,2S)-2, CH_2_ bridge] acted as a ratiometric probe toward Ag(I) concentration.

When the OPE unit was modified with sulfoxides at the end positions, oxophilic metal cations (Ca^2+^, Sc^3+^, and Zn^2+^) interacted with oxygen atoms, promoted the folding of OPE to form helical conformation, and turned on the chiroptical properties (Reiné et al., 2018). Meanwhile, the helical conformation avoided aggregation induced emission quenching of the fluorophore itself. Consequently, the metal complexes exhibited both glum values and high Φ_Fl_. A representative example was the Sc^3+^ complex, of which the g_lum_ and Φ_Fl_ were −7.1 × 10^−3^ and 0.38, respectively.

## pH

**Figure 10:**
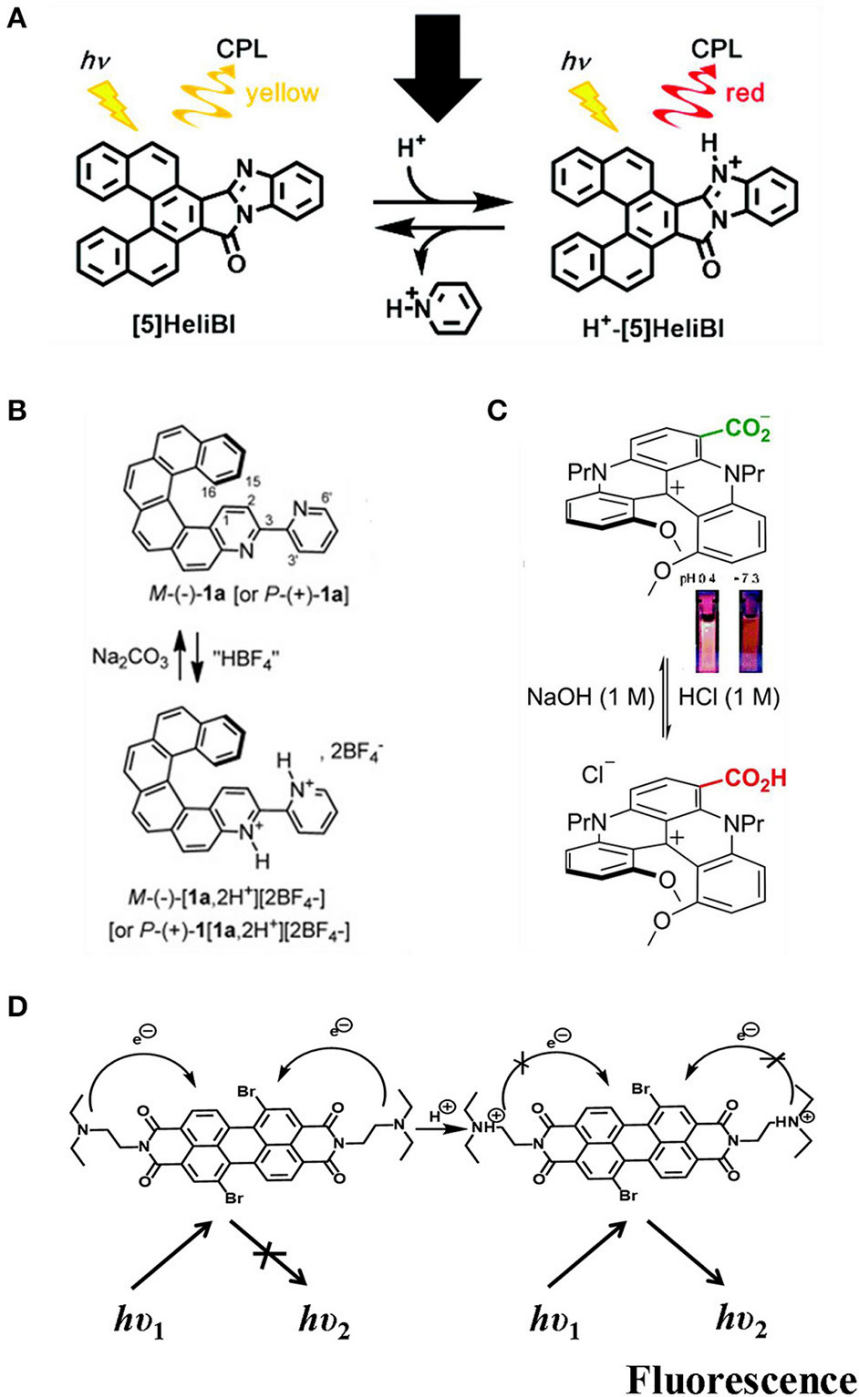
**(A,B)** pH induced switchable CPL based on pyridine N- atom. **(C)** deprotonation of aromatic carboxylic acid. **(D)** protonation of tertiary amine. **(A)** Reproduced with permission (Sakai et al., 2016). Copyright 2016, Royal Society of Chemistry. **(B)** Reproduced with permission (Saleh et al., 2015). Copyright 2015, John Wiley and Sons. **(C)** Reproduced with permission (Pascal et al., 2016). Copyright 2016, Royal Society of Chemistry. **(D)** Reproduced with permission (Han D. et al., 2018). Copyright 2018, Royal Society of Chemistry.

Manipulating pH value has been demonstrated to be a broadly applicable tool to tune both the wavelength and the intensity of CPL. The wavelength shift was achieved by protonation of N-heterocycle-containing chromophores or deprotonation of acidic protons. The intensity tune was exemplified by tuning the competing non-emissive pathways. Moreover, both changes could be facilely reversed by reverting to the original pH by adding appropriate amounts of acid or base. The CPL color of benzimidazole-fused [5]carbohelicene ([5]HeliBI) (Figure 10A) can be switched from yellow (570 nm) to red (650 nm) by protonation with trifluoroacetic acid to form (H^+^- [5]HeliBI) (Sakai et al., 2016). Both yellow and red CPL show high g_lum_ values in the CH_2_Cl_2_ solution, which were estimated to be ~9.45 × 10^−3^ and ~5.92 × 10^−3^, respectively. Moreover, the process was reversible by deprotonation with pyridine. Similar reversible CPL emission tuned by acid/base addition (HBF_4_, Na_2_CO_3_/NEt_3_) was observed for both P- and M- 3-(2-pyridyl)-4-aza[6]helicene (Saleh et al., 2015). Bathchromic emission from 426 to 590 nm was observed after dual protonation, while the quantum yield, lifetime, and g_lum_ value (~10^−3^) remained almost the same (Figure 10B). For diaza [4] helicene functioned with a carboxylic acid group, lowering the pH from 7.3 to 0.4 resulted in protonation of the zwitterion form to the protonated from (Pascal et al., 2016), and the fluorescence emission was shifted from 709 nm (acetonitrile, QY~0.01%) for to 654 nm (QY ~0.29%) (Figure 10C). Differently, no CPL signal was detected for the former, while CPL signal (600 nm) was turned on for the later with a g_lum_ value of ~5 × 10^−4^. Thus, an off-on CPL switch was achieved by increasing/lowering the acidity of the system. Turn-on of CPL by lowering pH was also observed for a co-assembled gel of achiral perylene bisimide dye and N,N'-bis(octadecyl)-L/D-Boc glutamic diamide gelator (Figure 10D), which was a result of chiral transfer from the chiral part to PBI and inhibited photon induced charge transfer in the PBI unit (Han D. et al., 2018). The CPL sign was the same as the chirality of the chiral gelator, and the bright yellow emission was centered at 558 nm. The g_lum_ was calculated to be a moderate ~9 × 10^−3^. When the co-gel was exposed to a basic atmosphere, such as ammonia, the CPL signal could be reversibly switched off. Thus, an on-off CPL switch was obtained by applying the co-gel to alternating acidic and basic conditions.

**Figure 11:**
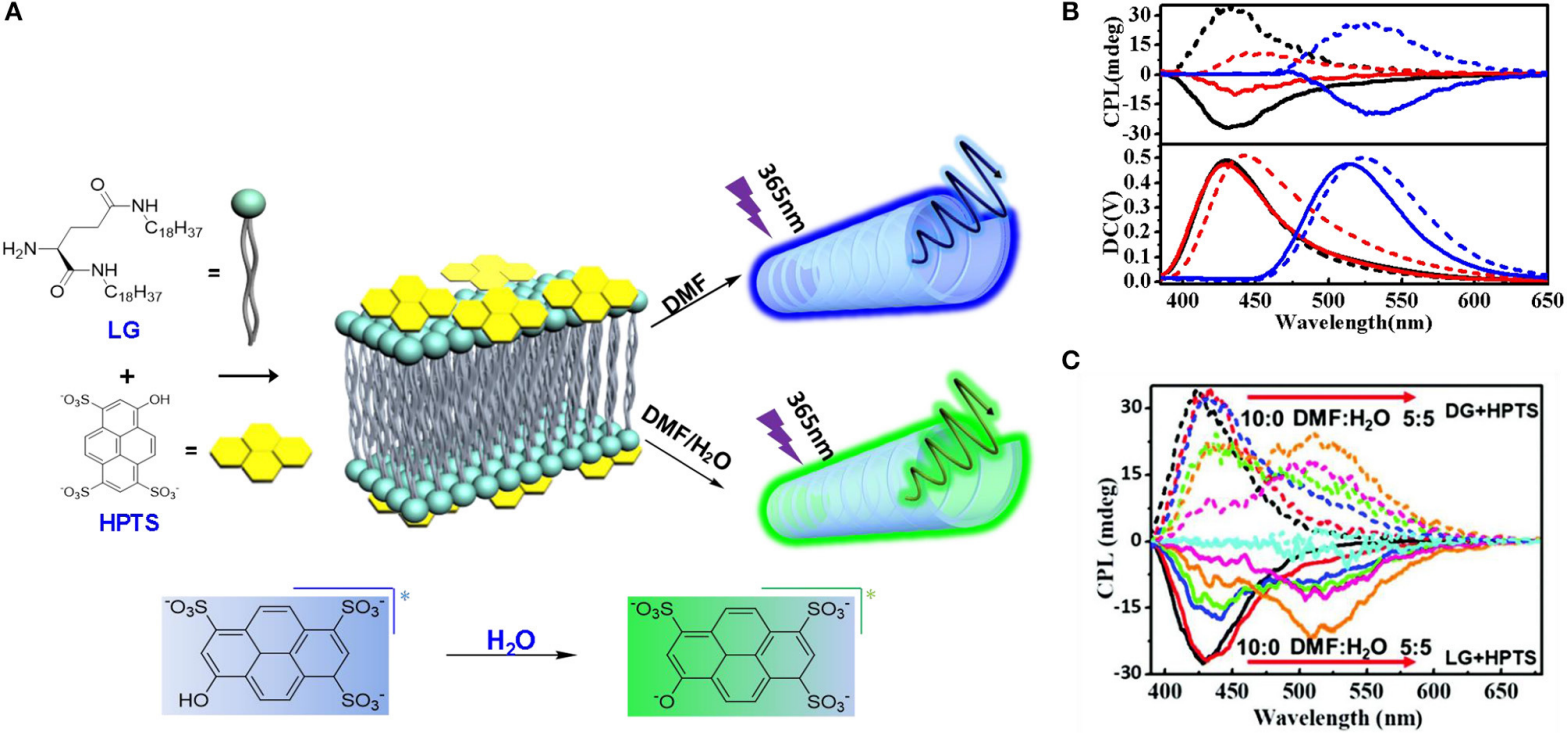
**(A)** Chemical structure of LG and HPTS, and the schematic demonstration about CPL dependence on solvent by deprotonation (RO–). **(B)** CPL spectra of LG/HPTS (DMF/H_2_O = 9 : 1) gels (black line) and LG/HPTS gels with acid (red line) and base (blue line). Solid lines refer to LG/HPTS and dotted lines represent the DG/HPTS system. **(C)** CPL spectra of LG/HPTS (dash) and DG/HPTS (line) supramolecular gels in mixed solvents of N,N-Dimethylformamide (DMF) and water (H_2_O). The measurements were carried out in the solutions with a concentration of ~1.23 × 10^−4^ M at room temperature, under the excitation of 370 nm. Reproduced with permission (Fan et al., 2019). Copyright 2019, Royal Society of Chemistry.

Besides the above-mentioned cases using Lewis acid/base and Brönsted acid/base activation, a photo-acid functionalized dye also showed pH dependent emission behavior. In the co-assembled gel of non-emissive photoacid 8-hydroxy-1,3,6-pyrenetrisulfonate (HPTS) and chiral amino-terminated dialkyl glutamide (LG) (Figure 11), the emission wavelength and the g_lum_ value was modulated by the composition of solvent or acid/base additive (Fan et al., 2019). A mixed molar ratio of 1:10 of HPTS:LG assembled in DMF:H_2_O resulted in nanotube structure, with the components arranged in a lamellar motif. CD signals in the same range of absorption (250–450 nm) retained the chirality from the gelator, which proved that chirality transfer occurred from the gelator to the photoacid. Upon excitation in a proton-accepting environment (mixed solvent of DMF:H_2_O ratio <7:3, or basic condition), excited-state induced proton transfer from the photoacid to the environment shifted the CPL signal from 420 nm (pristine HPTS) to 520 nm (deprotonated HPTS). Meanwhile, the g_lum_ of the green emission was increased to 10^−3^.

## Controlled Assembly Switchable by Solvent, Temperature, and Other Miscellaneous Factors

CPL materials usually showed higher g_lum_ values from aggregates or assembled states than from the monomers, and manipulation of the assembly behavior resulted in tunable CPL performances. Broadly applicable manipulating factors included solvent, temperature, and mechanical forces.

**Figure 12:**
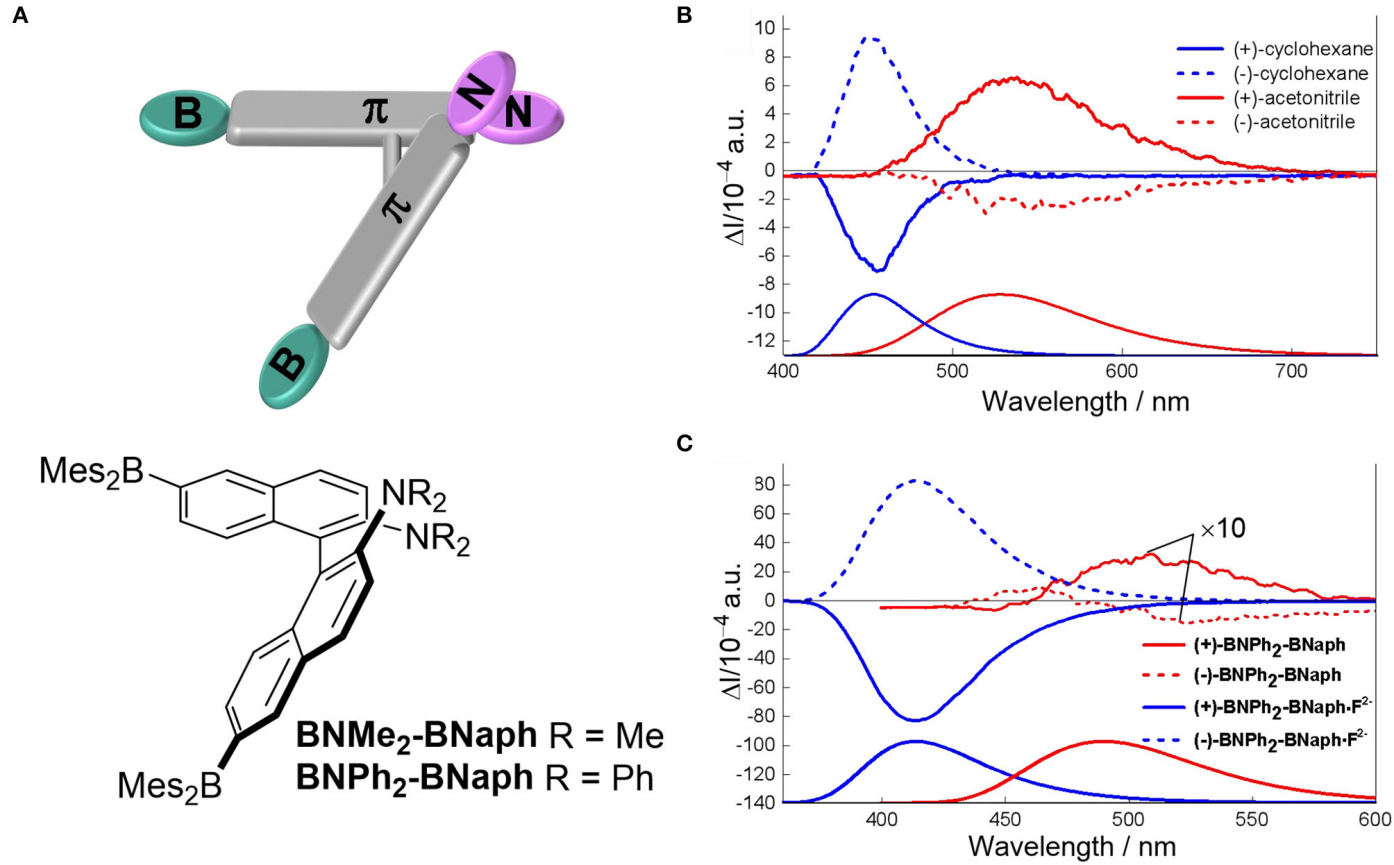
**(A)** Illustration and chemical structure of chiral BNPh_2_-BNaph. **(B)** CPL (top) and fluorescence (bottom) spectra of BNPh_2_-BNaph in cyclohexane (blue) and MeCN (red), condition: 1.23 × 10^−4^ M, λ_ex_ = 340 nm. **(C)** CPL (top) and fluorescence (bottom) changes of BNPh_2_-BNaph upon addition of F^−^ (tetrabutylammonium fluoride, 20 equiv). The measurements were carried out in THF solution (~1.23 × 10^−4^ M), under the excitation of 320 nm. Reproduced with permission (Sun Z. et al., 2019). Copyright 2019, John Wiley and Sons.

**Figure 13:**
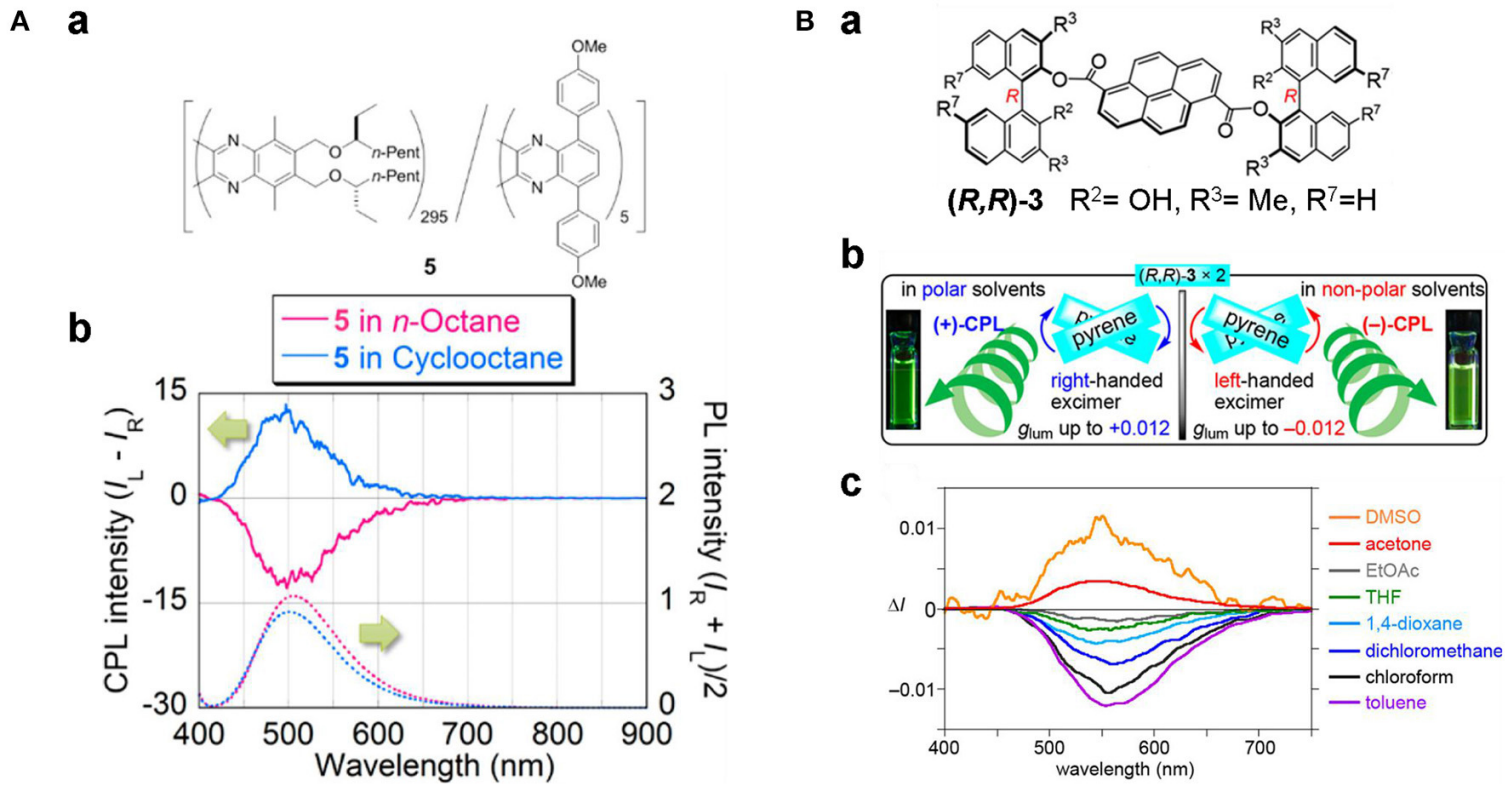
**(A)** (a) Chemical sketch of polymer 5, and (b) CPL (upper) and PL (lower) spectra of **5** in n-octane (red) and cyclo-octane (blue) (2.70 × 10^−2^ g/L). **(B)** (a, b) Chemical structures and CPL illustration of (*R,R*)-**3**; (c) CPL spectra of **(*R,R*)-3** in different solvents. The solutions (~4.0 × 10^−3^ M) were excited at 355 nm. **(A)** Reproduced with permission (Nishikawa et al., 2017). Copyright 2017, American Chemical Society. **(B)** Reproduced with permission (Takaishi et al., 2020). Copyright 2020, American Chemical Society.

Solvents with varied polarity, hydrogen-bonding capabilities and solubility have been used to tune the electronic structure through intramolecular charge transfer (ICT), the assembly behaviors, and the formation of excimers, respectively. Modulating the intramolecular charge transfer process of axial chiral triarylboranes resulted in switchable CPL behaviors. For 2,2′-bis(diphenylamino)-6,6′-bis(dimesityl-boryl)-1,1′-binapthyl with a D-π-A structure (Sun Z. et al., 2019), increasing solvent polarity from cyclohexane (455 nm) to MeCN (521 nm) showed an obvious red-shifted emission from 440 to 512 nm (Figure 12). Correspondingly, reversal of CPL sign was observed for the (+)-isomer in cyclohexane (negative signal) and in MeCN (positive signal). Similar sign inversion was also detected by using an excess amount of fluoride ions (F^−^), which complexed with the boron atoms to greatly enhance the emission intensity and asymmetry (|g_lum_| ~10^−2^). Handedness reversion by changing solvents was also observed due to reversal of the orientation of the helix. For a random polymer containing achiral fluorescent 5,7-bis(4-methyloxyphenyl)quinoxaline and chiral units modified with (S)-3-octyloxymethyl groups (Nishikawa et al., 2017), solvent change from n-octane to cyclooctane resulted in conformation change of the polymer from pure M- to P-helical, and the correspondent CPL shifted from right handedness to left handedness (Figure 13A). Moreover, the change of conformation was determined only by the chiral moiety, and the emission color can be tuned by modifying the luminescent part with different substituents. Thus, the polymer can be tuned orthogonally to prepare CPL materials with convertible handedness and variable emission colors. Furthermore, solvent tunable handedness was achieved for pyrene incorporated axially chiral 2-hydroxy 3, 3′-dimethylbinathyls with carefully designed substituents (Figure 13B). Highly polarized negative excimer emission in non-polar toluene (538 nm, g_lum_ ~-0.012) was reversed to right-handedness in polar DMSO (520 nm, g_lum_ ~0.012) (Takaishi et al., 2020). The sign switch was attributed to the reduced intermolecular H-bonding in the polar solvents and corresponding lower population of the left-handed excimer. This hypothesis was confirmed further by lowering the temperature. This resulted in the stronger negative emission being observed in toluene and lower positive emission being observed in acetone.

Along with switchable CPL behaviors in pure solvent, a ratio change between good solvent and poor solvent (mixture of THF and H_2_O) has also been reported to produce tunable CPL. For a 1,8-naphthalimide fluorophore linked with chiral 1,2-diaminocyclohexane (DACH) (Sheng et al., 2016), excimer-type CPL with large g_lum_ (~10^−2^) was observed in THF or mixture of H_2_O and THF with <90% water. Upon increasing the water content above 90%, the assembly changed from an irregular form to an ordered aggregate, and the CPL sign was reversed. Similar THF/water ratio dependent assembly and CPL performance were observed for R- or S-SPAn (Ma K. et al., 2019). With increasing water ratio, the orderly aggregates changed from 0D nanoshperes (THF/H_2_O ~50:50), 2D flake (THF/H_2_O ~85%) to 3D nanoflakes (THF/H_2_O ~90%). Meanwhile, the emission wavelength shifted from 420 nm (Φ_FL:_ 0.6), 432 nm (Φ_FL:_ 0.04) to 460 nm (Φ_FL:_ 0.13). The related g_lum_ value increased from the value of 10^−4^, 7.2 × 10^−3^, to 2.9 × 10^−2^, inferring favor of high g_lum_ with larger ordered structures.

**Figure 14:**
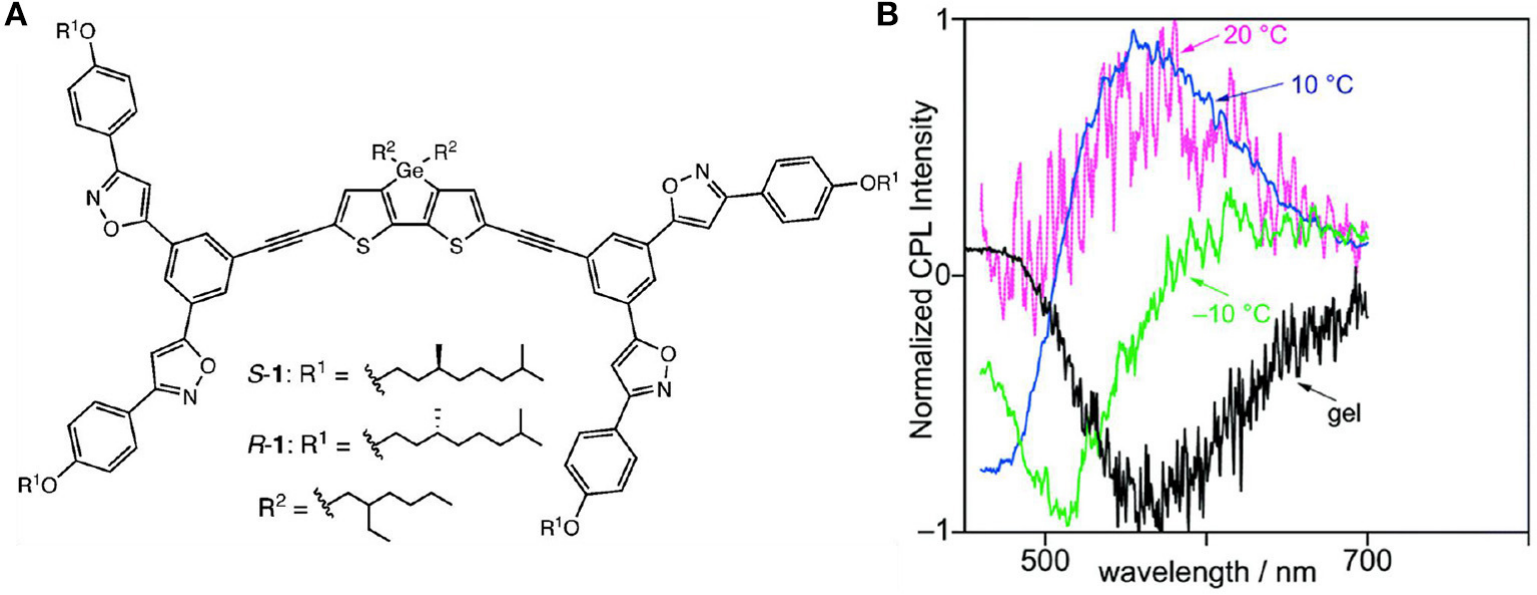
**(A)** Skeleton of DTG possessing phenylisoxazoles and substituents. **(B)** CPL plots of S-1 from the methylcyclohexane solution (~4.9 × 10^−4^ M) to gel, under the excitation of 420 nm. Reproduced with permission (Hirano et al., 2019). Copyright 2019, Royal Society of Chemistry.

The CPL behaviors were influenced by temperature through cooling to form ordered chiral assemblies or through heating to dissolve the assembly, which was utilized to tune either the CPL handedness or the emission wavelength. Upon lowering the temperatures, two-stage cooperative assembly of dithienogermole (DTG) molecules driven by dipole-dipole interaction of chiral phenylisoxazoles pendants was observed in methyl-cyclohexane (MCH), including a first nucleation stage and a later elongation stage. Correspondingly, CPL signals with different emission wavelengths and inverted handedness were observed (Hirano et al., 2019). When the temperature was initially dropped from 40 to 20°C, a weak positive CPL appeared at 566 nm (Figure 14). Further cooling to 10°C resulted in increased CPL at 566 nm, and a new negative signal at 472 nm was observed. After further cooling to −10°C, the positive signal disappeared, and only a negative signal at 514 nm was detected. Thus, the temperature dropping from 20 to −10°C fully inverted a positive signal at 566 nm (g_lum_ ~8 × 10^−4^) to a negative signal at 514 nm (g_lum_ ~-3 × 10^−4^). Similar sign change was also observed in the CD signals for both the S- and R- enantiomers, which suggested the assembly behavior dominated the chirality in both the ground and excited states. Thus, chirality inversion was achieved by controlled assembly at different temperatures. Similarly, L-glutamide-functionalized phenyl anthracene (g-PA) showed solvent- and cooling-rate-dependent CPL signals (Jintoku et al., 2015). In pure THF, no CPL signal was observed. In a mixture of n-hexane/THF (50:1), the organic gel showed clear excimer-type CPL signal at 526 nm, and the g_lum_ value is ~3.2 × 10^−3^. Thus, the gel state enhanced chirality transfer from the L-glutamide unit to the excimer of anthracene. As the temperature elevated from 10 to 60°C, excimer emission was changed to monomer emission (426 nm) with decreasing g_lum_. Moreover, the slower the reversed cooling process, the higher ratio of the excimer CPL at 10°C.

**Figure 15:**
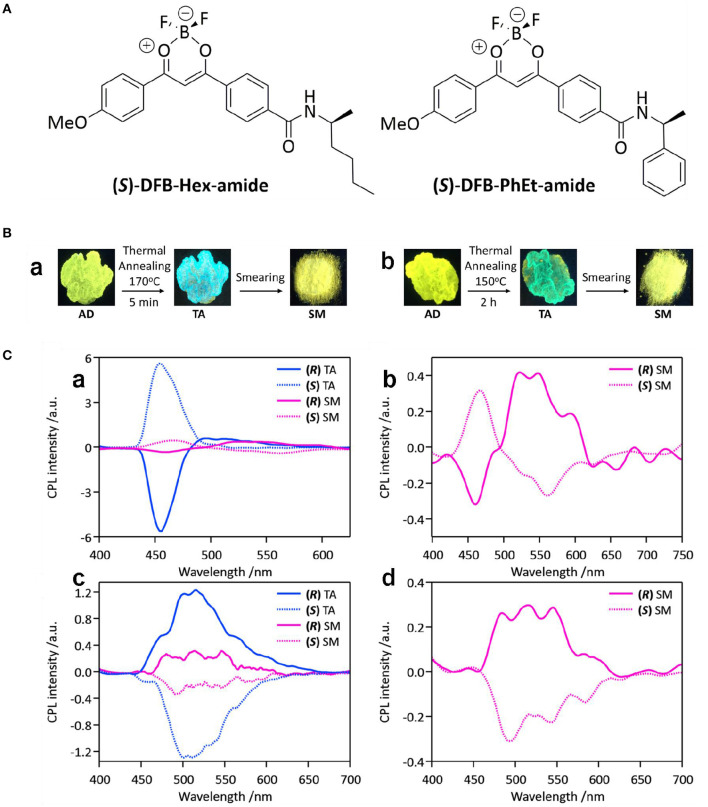
**(A)** Chemical structures of (S)-DFB-Hex-amide and (S)-DFB-PhEt-amide. **(B)** Photographs of the samples obtained from deposition (AD), annealing (TA) and smearing (SM) for (S)-DFB-Hex-amide (a) and (S)-DFB-PhEt-amide (b). Under the excitation of 365 nm. **(C)** CPL spectra of (R/S)-DFB-Hex-amide (a and b) and (R/S)-DFB-PhEt-amide(c and d). Solid-state samples were deposited on paper. Blue color indicates the thermally annealed (TA) samples, while the pink color represents the mechanically smeared samples. (R) and (S) isomers are plotted using solid and dashed lines, respectively. Reproduced with permission (Louis et al., 2019). Copyright 2019, Royal Society of Chemistry.

Moreover, CPL performance switched with mechanical triggers, and oxidation anions and co-assembled achiral isomers were reported. Mechanical mixing of difluoro-boron β-diketonate complexes with chiral amide ligands (DFB-Hex-amide) triggered CPL sign inversion (Louis et al., 2019). For the crystalline state obtained from thermal annealing, strong emission appeared in the blue-green range (475 nm, Φ_FL_ ~0.10–0.13), and the g_lum_ value reached the level of ~10^−2^ (Figure 15). After applying a shearing stress, the CPL signs for the two signals at 450 and 550 nm (Φ_FL_~0.35) were inverted, and the g_lum_ value (500–550 nm) dropped to 3 × 10^−3^. By referring to structural analogs and the fluorescence lifetime measurements, the signal at 450 nm was ascribed to the remaining microcrystals in the smeared sample, while the signal at 500–550 nm was assigned to the excimers. A CPL sign dependent on the amount of hypochlorite ion (ClO^−^) present was observed for a chiral gel composed of an achiral phenothiazine derivative and a gelating chiral glutamic lipid (PTD-Z) through step-wise oxidation of the sulfur atom in the phenothiazine ring (Gong et al., 2019). Initial titration experiments were carried out on a solution of PTD-Z (10 μM, mixed CH_3_CN/CHCl_3_). Addition of ClO^−^ from 0–80 μM was accompanied with emerging absorption at 375 nm and blue-shifted emission from 625 to 498 nm. Further addition of ClO^−^ to 10 equivalents of PTZ-D resulted in the formation of a sulfone. Structurally, the original left-handed helicoid structure of the PTZ-D gel changed to a right-handed structure. As a result, the original negative CD signal at 438 nm gradually shifted to a positive signal at 460 nm. Correspondently, the negative CPL signal initially decreased, and then reversed to positive. The change was ascribed to the different dihedral angles related to the phenothiazine unit before and after oxidation. In Feng's work, co-assembled supramolecular gels composed of C2-symmetric hydrogelators (LPF and DPF) and achiral naphthylamine isomers showed inversed supramolecular chirality and CD/CPL signals when the amine groups of the isomers shifted from the α- position to the β- position (Yang et al., 2019). Along with the sign inversion, the CPL signals exhibited high |glum| values in the range of 5.62 × 10^−3^-8.74 × 10^−3^. Furthermore, the inversion was studied from the intermolecular hydrogen bonds and π-π stacking between the chiral and non-chiral components, which served as a rare example of achiral isomers used to form switchable CPL-active materials.

## Conclusions and Perspectives

Various CPL organic materials have been developed through tailoring either the molecular structures or their assembly behavior. Those with switchable CPL properties have been reviewed here, which possess considerable potential for applications in information processing and as intelligent sensors. The switchable behaviors have been classified based on different stimuli, including photo-irradiation, host–guest interaction, metal ions, pH, solvent, temperature, etc. Photoactive CPL switches have been developed mainly from photochemical active units, such as covalently linked photochromic diaryethene/spiropyran and chiral units or by chiral assembly of photo-isomerizable cyanostilenes. Recently, a novel strategy based on photophysical excitation of selected excited states has been applied to achieve convertible CPL and CP-OURTP. For host–guest interaction-based switches, cryptand molecules with tunable size and adjustable binding affinity have been used to control both the formation of chiral inclusion pairs and to modulate the chirality transfer process in-between the components. Recent progress has come from a robust on-off CPL shuttle based on a single molecular machine. Metal ions serve as a versatile methodology with which to switch the CPL behaviors by forming highly directional and rigid complexes, which can dictate related electronic band structures, molecular conformations and helical orientations. Thus, multiple metal ions have been applied to reach CPL switches in emission color, intensity, and handedness. A majority of acid-base active CPL switches tune the emission wavelength by protonation/deprotonation process, and a recent on-off switch works by governing the protonation induced electron transfer. Solvent and temperature based CPL switches make impacts by the facile operation. The switch processes work usually by controlling the assembly process, through tuning the solvent-solute interaction forces by tuning the polarity of the solvents, or by controlled cooling. Besides those widely applied stimuli, other methods not mentioned here make use of anions (Maeda et al., 2011; Zhao Z. H. et al., 2019), energy transfer (Feng et al., 2018; Sun M. J. et al., 2019), and stoichiometry (Li et al., 2019).

To sum up, various types of stimuli for CPL switches work by either tuning molecular/electronic structures to change the related dynamic assembly behaviors or tuning external interactions in-between chiral and achiral components/moieties. Meanwhile, the many switch modes can be classified into two general fashions, simple on or off CPL switches or switching between/among multiple emissive CPL states. Up till now, the former have been in the majority. Meanwhile, a large part of the CPL switches rely on the synchronous luminescence switch. Thus, CPL switches capable of switching among multiple emissive states and CPL switches independent on the luminescence switch are still less explored and under demand. Hopefully, the methodologies discussed in the review will help to promote further research.

## Author Contributions

YG and TH initiated the project. YG, CR, XL, and TH searched the data and wrote, revised, and completed the manuscript. All authors contributed to the article and approved the submitted version.

## Conflict of Interest

The authors declare that the research was conducted in the absence of any commercial or financial relationships that could be construed as a potential conflict of interest.

## References

[B1] Chen J. Chen Y. Zhao L. Feng L. Xing F. Zhao C. (2019). G-quadruplex DNA regulates invertible circularly polarized luminescence. J. Mater. Chem. C 7, 13947–13952. 10.1039/c9tc04508b

[B2] David A. H. G. Casares R. Cuerva J. M. Campana A. G. Blanco V. (2019). A [2]rotaxane-based circularly polarized luminescence switch. J. Am. Chem. Soc. 141, 18064–18074. 10.1021/jacs.9b07143 31638802 PMC6975276

[B3] Fan H. Jiang H. Zhu X. Guo Z. Zhang L. Liu M. (2019). Switchable circularly polarized luminescence from a photoacid co-assembled organic nanotube. Nanoscale 11, 10504–10510. 10.1039/c9nr01959f 31115419

[B4] Feng H.-T. Gu X. Lam J. W. Y. Zheng Y.-S. Tang B. Z. (2018). Design of multi-functional AIEgens: tunable emission, circularly polarized luminescence and self-assembly by dark through-bond energy transfer. J. Mater. Chem. C 6, 8934–8940. 10.1039/c8tc02504e

[B5] Gong J. Yu M. Wang C. Tan J. Wang S. Zhao S.. (2019). Reaction-based chiroptical sensing of ClO(-) using circularly polarized luminescence via self-assembly organogel. Chem. Commun. 55, 10768–10771. 10.1039/c9cc05245c 31432821

[B6] Guo Y. Han Y. Chen C. F. (2019). Construction of chiral nanoassemblies based on host–guest complexes and their responsive CD and CPL properties: chirality transfer from 2,6-helic[6]arenes to a stilbazolium derivative. Front. Chem. 7:543. 10.3389/fchem.2019.00543 31428601 PMC6688524

[B7] Han D. Han J. Huo S. Qu Z. Jiao T. Liu M.. (2018). Proton triggered circularly polarized luminescence in orthogonal- and co-assemblies of chiral gelators with achiral perylene bisimide. Chem. Commun. 54, 5630–5633. 10.1039/c8cc02777c 29774353

[B8] Han J. Guo S. Lu H. Liu S. Zhao Q. Huang W. (2018). Recent progress on circularly polarized luminescent materials for organic optoelectronic devices. Adv. Opt. Mater. 2010:1800538 10.1002/adom.201800538

[B9] Hashimoto Y. Nakashima T. Shimizu D. Kawai T. (2016). Photoswitching of an intramolecular chiral stack in a helical tetrathiazole. Chem. Commun. 52, 5171–5174. 10.1039/c6cc01277a 26996611

[B10] Hirano K. Ikeda T. Fujii N. Hirao T. Nakamura M. Adachi Y.. (2019). Helical assembly of a dithienogermole exhibiting switchable circularly polarized luminescence. Chem. Commun. 55, 10607–10610. 10.1039/c9cc05253d 31424063

[B11] Homberg A. Brun E. Zinna F. Pascal S. Gorecki M. Monnier L.. (2018). Combined reversible switching of ECD and quenching of CPL with chiral fluorescent macrocycles. Chem. Sci. 9, 7043–7052. 10.1039/c8sc02935k 30310624 PMC6137439

[B12] Imai Y. Yuasa J. (2019). Off-off-on chiroptical property switching of a pyrene luminophore by stepwise helicate formation. Chem. Commun. 55, 4095–4098. 10.1039/c9cc01138b 30887987

[B13] Isla H. Srebro-Hooper M. Jean M. Vanthuyne N. Roisnel T. Lunkley J. L.. (2016). Conformational changes and chiroptical switching of enantiopure bis-helicenic terpyridine upon Zn(2+) binding. Chem. Commun. 52, 5932–5935. 10.1039/c6cc01748g 27054507 PMC4840045

[B14] Ito H. Sakai H. Okayasu Y. Yuasa J. Mori T. Hasobe T. (2018). Significant Enhancement of absorption and luminescence dissymmetry factors in the far-red region: a Zinc(ii) homoleptic helicate formed by a pair of achiral dipyrromethene ligands. Chemistry 24, 16889–16894. 10.1002/chem.201804171 30179282

[B15] Ji L. He Q. Niu D. Tan J. Ouyang G. Liu M. (2019). Host–guest interaction enabled chiroptical photo-switching and enhanced circularly polarized luminescence. Chem. Commun. 55, 11747–11750. 10.1039/c9cc06305f 31513199

[B16] Jin Q. Chen S. Jiang H. Wang Y. Zhang L. Liu M. (2018). Self-assembly of amphiphilic schiff base and selectively turn on circularly polarized luminescence by Al(3). Langmuir 34, 14402–14409. 10.1021/acs.langmuir.8b03019 30398358

[B17] Jin X. Yang D. Jiang Y. Duan P. Liu M. (2018). Light-triggered self-assembly of a cyanostilbene-conjugated glutamide from nanobelts to nanotoroids and inversion of circularly polarized luminescence. Chem. Commun. 54, 4513–4516. 10.1039/C8CC00893K 29561030

[B18] Jintoku H. Kao M.-T. Guerzo A. D. Yoshigashima Y. Masunaga T. Takafujiac M. (2015). Tunable Stokes shift and circularly polarized luminescence by supramolecular gel. J. Mater. Chem. C 3, 5970–5975. 10.1039/C5TC00878F

[B19] Li H. Li H. Wang W. Tao Y. Wang S. Yang Q.. (2020). Stimuli-responsive circularly polarized organic ultralong room temperature phosphorescence. Angew. Chem. Int. Ed. 59, 4756–4762. 10.1002/anie.201915164 31901181

[B20] Li P. Lu B. Han D. Duan P. Liu M. Yin M. (2019). Stoichiometry-controlled inversion of circularly polarized luminescence in co-assembly of chiral gelators with an achiral tetraphenylethylene derivative. Chem. Commun. 55, 2194–2197. 10.1039/c8cc08924h 30702088

[B21] Louis M. Sethy R. Kumar J. Katao S. Guillot R. Nakashima T.. (2019). Mechano-responsive circularly polarized luminescence of organic solid-state chiral emitters. Chem. Sci. 10, 843–847. 10.1039/c8sc04026e 30774879 PMC6345345

[B22] Ma J. L. Peng Q. Zhao C. H. (2019). Circularly polarized luminescence switching in small organic molecules. Chemistry 25, 15441–15454. 10.1002/chem.201903252 31550061

[B23] Ma K. Chen W. Jiao T. Jin X. Sang Y. Yang D.. (2019). Boosting the circularly polarized luminescence of small organic molecules via multi-dimensional morphology control. Chem. Sci. 10, 6821–6827. 10.1039/c9sc01577a 31391904 PMC6657416

[B24] Maeda H. Bando Y. Shimomura K. Yamada I. Naito M. Nobusawa K.. (2011). Chemical-stimuli-controllable circularly polarized luminescence from anion-responsive pi-conjugated molecules. J. Am. Chem. Soc. 133, 9266–9269. 10.1021/ja203206g 21599014

[B25] Meng F. Li F. Yang L. Wang Y. Quan Y. Cheng Y. (2018). The amplified circularly polarized luminescence emission response of chiral 1,10 -binaphthol-based polymers via Zn(II)-coordination fluorescence enhancement. J. Polym. Sci. Pol. Chem. 29, 1282–1288. 10.1002/pola.29009

[B26] Miao W. Wang S. Liu M. (2017). Reversible quadruple switching with optical, chiroptical, helicity, and macropattern in self-assembled spiropyran gels. Adv. Funct. Mater. 27:1368 10.1002/adfm.201701368

[B27] Morcillo S. P. Miguel D. Alvarez de Cienfuegos L. Justicia J. Abbate S. Castiglioni E.. (2016). Stapled helical o-OPE foldamers as new circularly polarized luminescence emitters based on carbophilic interactions with Ag(i)-sensitivity. Chem. Sci. 7, 5663–5670. 10.1039/c6sc01808d 30034704 PMC6022022

[B28] Nishikawa T. Nagata Y. Suginome M. (2017). Poly(quinoxaline-2,3-diyl) as a multifunctional chiral scaffold for circularly polarized luminescent materials: color tuning, energy transfer, and switching of the CPL handedness. ACS Macro. Lett. 6, 431–435. 10.1021/acsmacrolett.7b00131 35610846

[B29] Niu D. Jiang Y. Ji L. Ouyang G. Liu M. (2019). Self-assembly through coordination and pi-stacking: controlled switching of circularly polarized luminescence. Angew. Chem. Int. Ed. Engl. 58, 5946–5950. 10.1002/anie.201900607 30821078

[B30] Ouyang G. Liu M. (2020). Self-assembly of chiral supra-amphiphiles. Mater. Chem. Front (4), 155-167. 10.1039/C9QM00571D

[B31] Pascal S. Besnard C. Zinna F. Di Bari L. Le Guennic B. Jacquemin D.. (2016). Zwitterionic [4]helicene: a water-soluble and reversible pH-triggered ECD/CPL chiroptical switch in the UV and red spectral regions. Org. Biomol. Chem. 14, 4590–4594. 10.1039/c6ob00752j 27139039

[B32] Pop F. Zigon N. Avarvari N. (2019). Main-Group-Based Electro- and Photoactive Chiral Materials. Chem. Rev. 119, 8435–8478. 10.1021/acs.chemrev.8b00770 30943018

[B33] Reine P. Justicia J. Morcillo S. P. Abbate S. Vaz B. Ribagorda M. (2018). Pyrene-Containing ortho-Oligo(phenylene)ethynylene foldamer as a ratiometric probe based on circularly polarized luminescence. J. Org. Chem. 83, 4455–4463. 10.1021/acs.joc.8b00162 29577727 PMC6145600

[B34] Reiné P. Ortuño A. M. Resa S. Álvarez de Cienfuegos L. Blanco V. Ruedas-Rama M. J.. (2018). OFF/ON switching of circularly polarized luminescence by oxophilic interaction of homochiral sulfoxide-containing o-OPEs with metal cations. Chem. Commun. 54, 13985–13988. 10.1039/c8cc08395a 30480686

[B35] Sakai H. Kubota T. Yuasa J. Araki Y. Sakanoue T. Takenobu T.. (2016). Protonation-induced red-coloured circularly polarized luminescence of [5]carbohelicene fused by benzimidazole. Org. Biomol. Chem. 14, 6738–6743. 10.1039/c6ob00937a 27319321

[B36] Saleh N. Moore B. 2nd Srebro M. Vanthuyne N. Toupet L. Williams J. A.. (2015). Acid/base-triggered switching of circularly polarized luminescence and electronic circular dichroism in organic and organometallic helicenes. Chemistry 21, 1673–1681. 10.1002/chem.201405176 25418503 PMC4801116

[B37] Sang Y. Han J. Zhao T. Duan P. Liu M. (2019). Circularly polarized luminescence in nanoassemblies: generation, amplification, and application. Adv. Mater. e1900110. 10.1002/adma.201900110 31394014

[B38] Sheng Y. Ma J. Liu S. Wang Y. Zhu C. Cheng Y. (2016). Strong and reversible circularly polarized luminescence emission of a chiral 1,8-naphthalimide fluorophore induced by excimer emission and orderly aggregation. Chemistry 22, 9519–9522. 10.1002/chem.201600891 27140195

[B39] Sun M. J. Liu Y. Zeng W. Zhao Y. S. Zhong Y. W. Yao J. (2019). Photoluminescent anisotropy amplification in polymorphic organic nanocrystals by light-harvesting energy transfer. J. Am. Chem. Soc. 141, 6157–6161. 10.1021/jacs.9b02055 30945852

[B40] Sun Z. B. Liu J. K. Yuan D. F. Zhao Z. H. Zhu X. Z. Liu D. H.. (2019). 2,2'-Diamino-6,6'-diboryl-1,1'-binaphthyl: a versatile building block for temperature-dependent dual fluorescence and switchable circularly polarized luminescence. Angew. Chem. Int. Ed. 58, 4840–4846. 10.1002/anie.201813320 30675973

[B41] Takaishi K. Iwachido K. Ema T. (2020). Solvent-induced sign inversion of circularly polarized luminescence: control of excimer chirality by hydrogen bonding. J. Am. Chem. Soc. 142, 1774–1779. 10.1021/jacs.9b13184 31909994

[B42] Wang F. Ji W. Yang P. Feng C. L. (2019). Inversion of circularly polarized luminescence of nanofibrous hydrogels through co-assembly with achiral coumarin derivatives. ACS Nano 13, 7281–7290. 10.1021/acsnano.9b03255 31150196

[B43] Yang L. Wang F. Auphedeous D. Y. Feng C. (2019). Achiral isomers controlled circularly polarized luminescence in supramolecular hydrogels. Nanoscale 11, 14210–14215. 10.1039/c9nr05033g 31317160

[B44] Zhang D. W. Li M. Chen C. F. (2020). Recent advances in circularly polarized electroluminescence based on organic light-emitting diodes. Chem. Soc. Rev. 49, 1331–1343. 10.1039/c9cs00680j 31999286

[B45] Zhao W. L. Li M. Lu H. Y. Chen C. F. (2019). Advances in helicene derivatives with circularly polarized luminescence. Chem. Commun. 55, 13793–13803. 10.1039/c9cc06861a 31603148

[B46] Zhao Z. H. Liang X. He M. X. Zhang M. Y. Zhao C. H. (2019). Triarylborane-based [5]Helicenes with full-color circularly polarized luminescence. Org. Lett. 21, 9569–9573. 10.1021/acs.orglett.9b03734 31710499

